# Patterns and tempo of *PCSK9* pseudogenizations suggest an ancient divergence in mammalian cholesterol homeostasis mechanisms

**DOI:** 10.1007/s10709-021-00113-x

**Published:** 2021-01-30

**Authors:** Barbara van Asch, Luís Filipe Teixeira da Costa

**Affiliations:** 1 Department of Genetics, Stellenbosch University, Private Bag X1, Matieland, 7602 South Africa; 2 Unit for Cardiac and Cardiovascular Genetics, Department of Medical Genetics, Oslo University Hospital, Nydalen, P.O. Box 4956, 0424 Oslo, Norway

**Keywords:** Cholesterol homeostasis, Molecular evolution, Gene loss, PCSK9, Human disease, Animal models

## Abstract

Proprotein convertase subtilisin/kexin type 9 (PCSK9) plays a central role in cholesterol homeostasis in humans as a major regulator of LDLR levels. PCSK9 is an intriguing protease in that it does not act by proteolysis but by preventing LDLR recirculation from endosomes to the plasma membrane. This, and the inexistence of any other proteolytic substrate but itself could suggest that PCSK9 is an exquisite example of evolutionary fine-tuning. However, the gene has been lost in several mammalian species, and null alleles are present (albeit at low frequencies) in some human populations without apparently deleterious health effects, raising the possibility that the *PCSK9* may have become dispensable in the mammalian lineage. To address this issue, we systematically recovered, assembled, corrected, annotated and analysed publicly available *PCSK9* sequences for 420 eutherian species to determine the distribution, frequencies, mechanisms and timing of *PCSK9* pseudogenization events, as well as the evolutionary pressures underlying the preservation or loss of the gene. We found a dramatic difference in the patterns of *PCSK9* retention and loss between Euarchontoglires—where there is strong pressure for gene preservation—and Laurasiatheria, where multiple independent events have led to *PCSK9* loss in most species. These results suggest that there is a fundamental difference in the regulation of cholesterol metabolism between Euarchontoglires and Laurasiatheria, which in turn has important implications for the use of Laurasiatheria species (e.g. pigs) as animal models of human cholesterol-related diseases.

**Supplementary information:**

The online version of this article (doi:10.1007/s10709-021-00113-x) contains supplementary material, which is available to authorized users.

## Introduction

A central role for Proprotein convertase subtilisin/kexin type 9 (PCSK9) in cholesterol homeostasis was first revealed by the discovery that mutations in the gene were responsible for cases of autosomal dominant hypercholesterolemia (Abifadel et al. 2003; Leren 2004; Timms et al. 2004). Elucidating the underlying mechanisms took several years and led to unexpected results. As a member of a protease family, PCSK9 was suspected to be involved in low-density lipoprotein receptor (LDLR) degradation, as confirmed by *PCSK9* over-expression experiments (Maxwell and Breslow 2004; Maxwell et al. 2005). Surprisingly, however, further experiments revealed that PCSK9 promotes LDLR degradation by a mechanism *independent* of its catalytic ability (which is only required for proper PCSK9 sub-cellular localization) (Seidah et al. 2003; Maxwell et al. 2005; Cameron et al. 2006; Lagace et al. 2006; McNutt et al. 2007; Li et al. 2007). In most proprotein convertases, autocatalytic cleavage between the prodomain and the mature protein is followed by a second cleavage *within* the prodomain, which is necessary for its release and activation of the protease (Anderson et al. 2002). In contrast, only the initial cleavage (Naureckiene et al. 2003) occurs in PCSK9, and the protein is secreted while still in a complex with the prodomain (Seidah et al. 2003; Benjannet et al. 2004). Furthermore, crystalographic studies provided an explanation for the tight association between PCSK9's prodomain and the mature protein, and suggested that the topology of that association blocks access to the catalytic triad, thus inhibiting further catalytic activity (Piper et al. 2007).

But how could a proteolytically inactive PCSK9 promote LDLR degradation? The puzzle was eventually solved with the realization that PCSK9 binds LDLR (particularly at its EGF-A domain) more tightly at low pH than at neutral pH, and the observation that the addition of PCSK9 to cell medium leads to a redistribution of LDLR from the plasma membrane to the lysosomes. These results implied that the binding of PCSK9 and its tightening at acidic pH prevent a conformational change in the LDLR that is necessary for its recycling from the endosomes to the plasma membrane, thus re-directing the LDLR to the lysosomes and leading to its degradation (Cunningham et al. 2007; Zhang et al. 2007; Fisher et al. 2007).

**Fig. 1:**
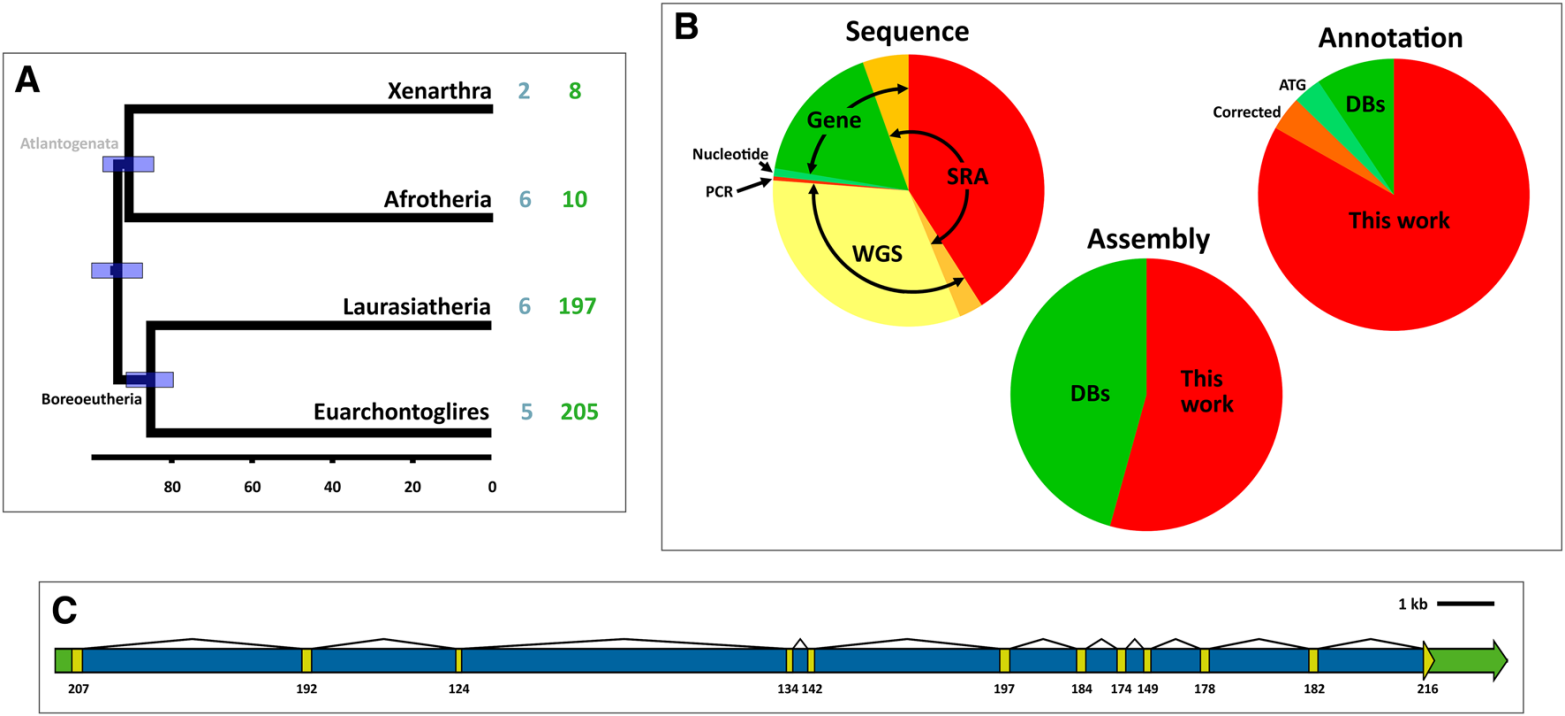
Overview of this study's scope. **a** Proposed phylogenetic tree of Eutheria (adapted from (Tarver et al. 2016)), indicating the number orders (blue) and species (green) included for each of the four supra-ordinal clades. Scale bar—millions of years before present. Node bars indicated 95% HPD for node dates. The Atlantogenata clade is shown in grey due to it being controversial. **b** Origin of species data used in this study. Sequence data was recovered from the Sequence Read Archive (SRA) database, whole genome sequencing project contigs (wgs), assembled genomes (Gene), Genbank's Nucleotide database (mRNA sequences from (Ding et al. 2007)) and PCR products. Assemblies and annotation were recovered from databases (DB) or were newly generated. Some database-recovered sequences required correction, while others had a questionable ATG assignment. **c** Typical structure of the PCSK9 gene. The structure of the human gene (from GenBank) is represented to scale, with coding sequences in yellow, 5′- and 3′-untranslated exonic sequences in green and introns in blue. The number of coding bases in each exon is indicated. Alternative transcripts have been identified in humans, but are not considered functional

A protease which has been fine-tuned into having only itself as a substrate and acting as something of a chaperone in directing a protein for degradation could be seen, from first principles, as an exquisite example of evolutionary refinement. However, non-functional alleles of *PCSK9* have been found in some human populations without apparent negative health effects, even in individuals carrying two null alleles (Cohen et al. 2005; Zhao et al. 2006; Hooper et al. 2007). Moreover, some studies suggested that *PCSK9* had undergone pseudogenization in other mammalian species (Ding et al. 2007; Cameron et al. 2008). Could it be that *PCSK9* had become evolutionarily unimportant in mammals, and on the path to pseudogenization? Taking advantage of the conserved synteny around the *PCSK9* locus from sharks to placental mammals, a preliminary survey was taken by counting the number of placental mammal entries in NCBI's Gene database for *PCSK9* and its flanking genes—*BSND* and *USP24*. At the time when this study was initiated there were 67 entries for PCSK9, 93 for *BSND*, and 94 for *USP24*, suggesting that a large percentage of species had lost PCSK9. Interestingly, most potential losses were found among Laurasiatheria, one of the four major clades of placental mammals. A similar—and rigorously supported—picture, based on a general analysis of gene losses in a panel of 62 mammals, was recently reported (Sharma and Hiller 2020). These results suggested that *PCSK9* may have become unimportant, at least in some mammalian lineages, but left many unanswered questions about the patterns of conservation and loss of *PCSK9* in placental mammals, particularly regarding the distribution, frequency, mechanisms and timing of pseudogenization events. Some of these questions were addressed in the present study by exhaustively recovering and performing comparative analyses of the eutherian *PCSK9* sequences available in the NCBI databases, including many species for which only raw sequence data was available. A total of 420 species from all eutherian orders was analysed, including 346 species for which *PCSK9* gene and pseudogene sequences were newly assembled or annotated (Fig. 1). This comprehensive dataset provides a general picture of the patterns and timing of pseudogenization events of *PCSK9* in placental mammals and the selective pressures acting upon the gene in different evolutionary lineages. The results suggest that the usefulness of mammal species as models of cholesterol metabolism-related diseases has more limitations than previously thought.

## Materials and methods

### Species selection

This study aimed at obtaining the broadest possible view of the *PCSK9* gene status and evolution in eutherian mammals, as provided by publicly available sequence data. All species for which sequences could be recovered from NCBI's RefSeq, Gene and whole-genome shotgun contig (wgs) databases, as well as a subset of sequences from the Sequence Read Archive (SRA) database, as of January 31st, 2019, were included. SRA-recovered data were selected so as to have a fair chance of recovering full or nearly full sequences, and included: (a) whole-exome exon capture data; (b) whole genome sequencing projects with at least 10× coverage; (c) RNA-seq data from mRNA isolated from liver or kidney (or "mixed tissues" including at least one of these). RNA-seq data searches excluded species belonging to clades where gene loss was thought to be the ancestral state, based on knowledge gathered from genomic data. When sequences were available for wild and domesticated individuals of the same species, the former were used. Some species were later excluded from the study due to poor coverage (most frequently observed in exon capture SRA data) or reiterated unavailability of SRA sequences. In the three members of the family Heteromyidae for which data was available, genomic data strongly suggested that a multiplication of the *PCSK9* gene had occurred. In every case, it was not clear how many *PCSK9*-like genes were present, or if the assembled contigs available were correct and/or represented the full complement of gene family members. Since it was beyond the scope of the present work to deal with those issues, Heteromyidae was also excluded. Finally, there was an instance in which searches against two supposedly different species (the hoary bamboo rat—*Rhyzomys pruinosus*—and the chinese zokor—*Eospalax fontanierii*) gave the exact same number of hits which displayed different run numbers but identical spot numbers. Since both samples had been run in the same lab at the same time, and comparison of a few sequences from spots with the same numbers showed that they were identical, it was concluded that there had been a mistake, and both sequences were excluded. On the other hand, several species that did not fulfill the initial criteria but were deemed important were included, either because they belong to clades absent or poorly represented in the "core set", or because they were instrumental in addressing specific questions raised during the study (e.g. how widespread gene losses are within Dipodidae). These included species for which data became available after January 31st, 2019, species with genomic data with lower coverage than the initial target or with mRNA data not from liver or kidney, as well as two species from which genomic DNA was PCR amplified and Sanger sequenced (see below). In total, sequences from 420 species were utilized for the analyses (ST1), including 226 cases of newly assembled sequences, 120 other cases of newly annotated sequences and 17 cases in which gene annotation was corrected (not including the 14 cases of questionable start codon assignment—see below) (Fig. 1).

### Data retrieval and generation

BLAST searches were performed against the relevant databases to recover wgs and SRA data. For Xenarthra, Afrotheria and Euarchontoglires, queries were typically genomic sequences of another species from the same order when searching wgs data. For searching SRAs, a mRNA sequence or a "mini-gene" sequence including only exons and 50 bases from each of their flanking introns of a species in the same family or the most closely related family available were used. For Laurasiatheria, using queries from the same order was insufficient, due to both intra-ordinal differences in exon losses and marked sequence divergence following pseudogenization. Given the low evolution rates in Cetacea, sequences from this sub-order were considered a reasonable proxy for the ancestral Laurasiatheria ones. The appropriate sequences (genomic or “mini-gene”) from *Orcinus orca* (a cetacean species for which database *PCSK9* sequences were complete and consistent) were therefore also used as an additional query.

DNA samples of the great gerbil (*Rhombomys opimus*) NHMO-DMA-412/1-D) and the northern birch mouse (*Sicista betulina*) (NHMO-DMA-423/1-D) were obtained from the NHMO DNA Bank at the Natural History Museum of Oslo and the corresponding sequences were deposited in Genbank (accession numbers MT680936 and MT680937). PCR amplifications were carried out in 15 μl volumes, using ~ 10 ng of genomic DNA, 20% 360 GC PCR enhancer (Applied Biosystems), 1 U (0.2 µl) AmpliTaq Gold 360 (Applied Biosystems) and 15 pmol of each primer (ST3)—first tested using mouse and Chinese hamster (*Cricetulus griseus*) (CHO cell line) DNA. Reactions were carried out either in Thermo Fisher Scientific's ammonium sulfate PCR buffer and 1.5 mM MgCl_2_ (for *R. opimus*' exon 3) or in Standard AmpliTaq Gold 360 PCR buffer and 2.83 mM MgCl_2_ (all other fragments). Cycling protocols included an initial 12 min at 95 °C, followed by 2 cycles of 95 °C for 30 s, 60 °C for 1 min and 72 °C for 1 min; 3 cycles of 95 °C for 30 s, 56 °C for 1 min and 72 °C for 1 min; 2 cycles of 95 °C for 30 s, 52 °C for 1 min and 72 °C for 1 min; and 38 (exons 5, 6, 8, 9 and 11) or 40 (exons 2, 3, 4 and 7) cycles of 95 °C for 30 s, 58 °C for 1 min and 72 °C for 1 min; and a final extension at 72 °C for 3 min. 2.5 μl of each PCR product were treated with 1.2 µl ExoProStar mix (GE Healthcare), and used in 15 μl sequencing reactions performed with the BigDye Terminator v3.1 cycle sequencing kit (Applied Biosystems) according to the manufacturer's instructions. Sequencing products were purified by 5 min centrifugation at 900×*g* through Sephadex G-50 (GE Healthcare) columns and run on a 3730xl DNA analyzer (Applied Biosystems). In cases where direct sequencing did not yield the clear sequences of the desired PCR products (due to the presence of multiple products), these were TA-cloned into an in-house developed vector (Pereira-Castro et al. 2012) and clones were sequenced with vector-binding primers.

### Sequence assemblies and corrections

UGENE (Okonechnikov et al. 2012) was used for some preliminary assemblies, while all the final assemblies were done in CLC Main Workbench v6.9 (Qiagen). SRA sequences were assembled against the relevant genomic or cDNA sequences from another species from the same family or the closest family available by read mapping and contig generation. Assemblies were visualized as multiple sequence alignments including also the reference and consensus sequences. Wgs contigs from the same species containing different parts of the *PCSK9* gene were also assembled in CLC Main Workbench, either automatically (when feasible) or "manually", guided either by BLAST comparisons between different contigs (run within CLC Main Workbench) or by sequence alignments with genomic sequences from another species (chosen as above), performed either with CLC Main Workbench or YASS (Noé and Kucherov 2005), run on its web server (https://bioinfo.lifl.fr/yass/yass.php). Data was recovered and assembled at different points over an extended period, so there were multiple instances in which a species gene was assembled in the course of this work before an assembled sequence became publicly available. Generally, the latter were preferred in the final analyses, but no systematic attempt was made to replace previously assembled sequences. In general, exonic sequence gaps in RefSeq and wgs data were covered (when feasible) by SRA data recovered using the approaches described above, eventually supplemented with SRA data searches using gap-surrounding sequences as queries (virtual genome walking). In some cases (e.g. the common bottlenose dolphin, *Tursiops truncatus*), such defects (or protein inactivation mutations) were detected in RefSeq sequences, but not in a contig derived from a separate genomic sequencing project and therefore the latter were used. Suspected protein-inactivating mutations, including large scale deletions, were also checked against SRA data when possible, and the genomic sequence was corrected to match the SRA data when they did not agree.

### Sequence annotation and analyses

Genomic sequence annotation of gene structure was done in CLC Main Workbench (Qiagen), either automatically copied from that of another species within a sequence alignment (eventually with subsequent small manual corrections) or (particularly in the case of gene remnant "archaeology") introduced manually, guided by BLAST comparisons run locally or by sequence alignments obtained with YASS. In cases where gene structure annotations in the RefSeq database deviated from the generally observed gene structure without clear justification (e.g. in the alpine marmot, *Marmota marmota* and other Sciuridae, as well as some primate species, for which an ATG upstream of the "typical" one had been annotated as the start codon), the annotation was altered to conform with the standard gene structure. A fasta file containing all 256 *PCSK9* gene coding sequences used in this study (the "corrected" sequence, i.e. without the 1 bp deletion in exon 12 and the 10 bp extension of exon 11, in the case of the nine-banded armadillo, *Dasypus novemcinctus*) is included in the supplemental materials.

DNA and protein sequence alignments were mostly performed using CLC Main Workbench or TranslatorX (Abascal et al. 2010) on its webserver (http://www.translatorx.co.uk/). Additional alignments, particularly to identify the breakpoints of large deletions affecting the *PCSK9* locus in groups where pseudogenization has occurred, were performed with YASS, run on its web server. Tests for relaxation (or intensification) of selection were performed with RELAX (Wertheim et al. 2015) on the Datamonkey (Pond et al. 2005; Weaver et al. 2018) webserver (www.datamonkey.org). In the case of functional genes, alignments used for RELAX analyses were restricted to species with complete sequences; in the analyses involving Perissodactyla, only codons present in all species of the order were included in the alignments. Identity values for residues outside the signal peptide were calculated with Geneious (Restricted) 8.1.5, using alignments restricted to species with complete sequences in the relevant regions, and from which insertions present only in a small number of species had been removed.

### Figures

Figures were generated using GIMP2, with elements obtained from CLC Main Workbench v6.9, Excel and FigTree v1.4.2.

## Results

### *PCSK9* function is conserved in all orders (but not all species) of Afrotheria and Xenarthra

**Fig. 2:**
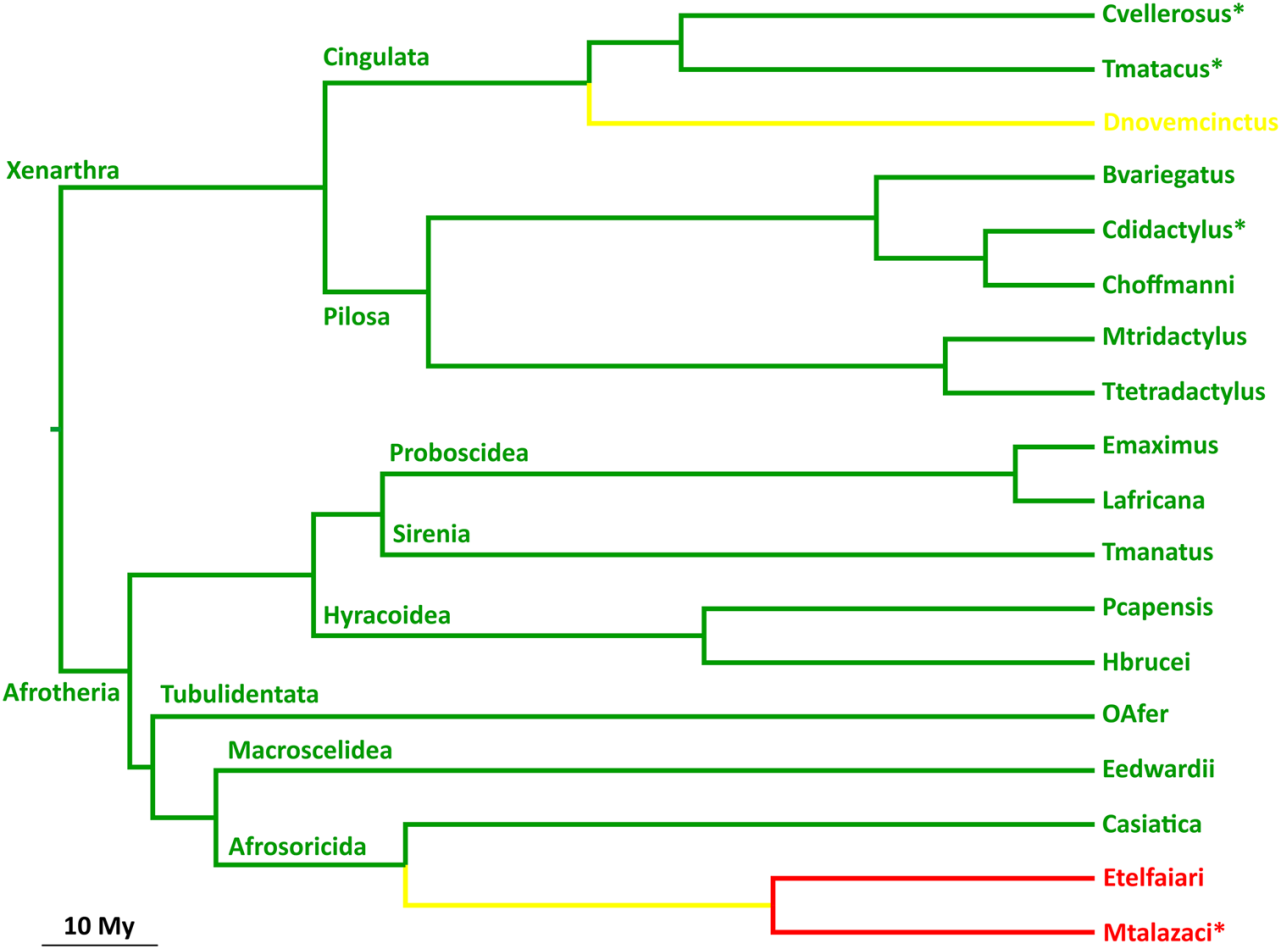
*PCSK9* gene function is preserved in all orders, but not all species, of Xenarthra and Laurasiatheria. Phylogenetic tree of the Xenarthra and Afrotheria species included in this study (adapted from (Poulakakis and Stamatakis 2010) and (Gibb et al. 2016)), with colors indicating gene status: green—functional; red—pseudogene; yellow—uncertain. *Incomplete sequence due to insufficient genomic coverage

There is strong evidence for a division of placental mammals in four major supra-ordinal clades: Laurasiatheria, Euarchontoglires, Afrotheria and Xenarthra (Murphy et al. 2001). The latter two—grouped in some analyses in the Atlantogenata clade—are comprised of a relatively small number of species originating in Africa (Afrotheria) and South America (Xenarthra). Complete or near complete (> 85%) *PCSK9* coding sequence data was only available for ten Afrotheria and eight Xenarthra species, likely reflecting the “species-poverty” of the two clades (ST1). Fifteen of these species, representing all orders of Afrotheria and Xenarthra, have apparently retained functional *PCSK9* genes (although locus coverage is incomplete in three cases) (Fig. 2). Evidence for gene functionality included conservation of splice junctions and internal exon sizes (with the exception of an in-frame 9-bp deletion in one species), absence of nonsense or frameshift mutations, and conservation of amino acids known to be functionally important, such as the catalytic triad and oxyanion hole residues, as well as the 25 cysteines in the catalytic and C-terminal domains (Abifadel et al. 2009) (SF1). Several species have deletions affecting the last 11 residues of the protein, including the deletion of all these residues in the two elephant species. However, these occur in one of the least conserved regions of PCSK9 (see below), and experimental evidence has shown that deletion of the last 12 residues of the human protein does not affect its function (Zhao et al. 2006). Thus, it is unlikely that the deletions observed in the C-terminus led to loss of function of PCSK9 in these species.

In contrast, *PCSK9* has clearly become non-functional in two Afrotheria species (the lesser hedgehog tenrec, *Echinops telfairi* and Talazac's shrew tenrec, *Microgale talazaci*), as both carry multiple protein-inactivating defects strongly supported by the raw sequence data, including stop codons and frameshift insertions and deletions. Interestingly, both species belong to the family Tenrecidae, raising the possibility that *PCSK9* pseudogenization is ancestral in tenrecs. However, there is no clear example of a shared inactivating alteration (the closest being a 1-bp deletion in one of the species overlapping with a 2-bp deletion in the other), so it is also formally possible that PCSK9 was independently lost in the two species.

Finally, the data for the nine-banded armadillo*,* one of the three Cingulata species analysed, is ambiguous. The *PCSK9* sequence displays multiple features suggesting that the gene is functional—conservation of a 12-exon structure, conserved internal exon sizes, absence of nonsense mutations, and conservation of known functionally important amino acids (SF1)—but the genomic reference sequence has a 1-bp deletion in exon 12, which should result in a non-functional protein. In the reference sequence, this alteration has been “compensated for” by a 3′ extension of exon 11 to include an additional 10 bp (SF2). As this entails the use on a non-canonical splice donor sequence, it is unlikely that it reflects the true transcript. A third possibility is that the 1-bp deletion is not actually present in the DNA of thenine-banded armadillo. Indeed, the genome of the species was sequenced at low coverage (6×), and the only available raw sequence covering the intron 11-exon 12 border does not have the 1-bp deletion in exon 12—although a single raw sequence is insufficient to settle this issue. An additional unusual feature of *PCSK9* in the nine-banded armadillo is that the stop codon is positioned 114 bp downstream relative to its human counterpart. However, it is also unlikely that this has an impact on protein function, since even larger C-terminal tags have been engineered into human PCSK9 without apparent functional impairment (Poirier et al. 2016). In summary, available sequence data suggests that the function of *PCSK9* has been preserved in members of all orders of Afrotheria and Xenarthra. However, it also shows that gene function has been lost in at least some afrotherian species (and possibly an entire family).

### Multiple independent events led to *PCSK9* inactivation in most Laurasiatheria

Compared to the paucity of sequence data available for Afrotheria and Xenarthra, Laurasiatheria and Euarchontoglires were abundantly represented. Almost two hundred laurasiatherian species could be analysed, providing a global picture of the patterns of gene conservation and loss within the clade. The first significant result obtained from the analysis of Laurasiatheria (and particularly of Cetartiodactyla) was the high level of discrepancy between annotated and primary sequence data. For example, the reconstitution of the genetic structure of *PCSK9* and its coding sequence was done for a total of 25 species of Cetacea (albeit with some limitations due to low genomic coverage in three of them). In contrast, only eight annotated entries were present in the Gene database, and five of these were found to contain sequence or annotation errors: exon 1 was either missing or incomplete in the minke whale (*Balaenoptera acutorostrata*), the narrow-ridged finless porpoise (*Neophocaena asiaeorientalis*), the sperm whale (*Physeter catodon*) and the common bottlenose dolphin due to sequence gaps, and although it was present in the reference sequence of the baiji (*Lipotes vexillifer*), it was not annotated as exon 1, perhaps due to annotation carry-over from a previous version of the genomic sequence where it was also missing. Furthermore, the genomic sequence of the common bottlenose dolphin also lacked exons 6 to 10, that of the minke whale displayed a mutation in the stop codon which would imply a 26 amino acid extension of the protein, and the coding sequence of the baiji was annotated as extending for the same additional 78 bp, even though a stop codon was indeed present at the typical position. On the other hand, analyses of whole genome sequence contigs and primary (i.e., Sequence Read Archive) data revealed a different picture. The 12 exons of *PCSK9* were indeed present in all species, there was no substitution generating a stop codon in the minke whale (not even in the actual specimen used for genomic sequence assembly), and the typical gene was expressed in the liver of all seven species for which mRNA data was available—including the minke whale, thus confirming the presence and transcription of exon 1 in that species. Important discrepancies between reference sequences and primary data for *PCSK9* also occurred in Camelidae and Suidae. There was a sequence gap indicated as being 20-bp long in exon 9 of the alpaca (*Vicugna pacus*), which would imply the presence of a 18-bp in-frame insertion; the 5′ end of exon 1's coding sequence was missing from the genomic sequences of both the dromedary (*Camelus dromedarius*) and the wild Bactrian camel (*Camelus ferus*), leading to the inclusion of an additional putative upstream exon; there was a frameshift mutation in exon 12 of the Bactrian camel*,* and two 1-bp deletions in exon 6 of the pig (*Sus scrofa*)—but in all cases primary sequence data could be used to demonstrate these were errors, and correct them. However, an unusual feature in *S. scrofa*'s *PCSK9* annotated sequence—a 39-bp 5′ extension of exon 4—was confirmed by primary data. This extension was caused by the combination of a G>A substitution in the last position of the ancestral intron 4, which inactivated the (already atypical) splice-acceptor, and an upstream mutation which created a splice-acceptor site (SF3). Primary sequence data revealed that both mutations were present in all six species of Suidae available, and also that the gene is expressed in pig liver and the novel splice-acceptor site is indeed used. Interestingly, neither of the mutations was present in the Chacoan pecari (*Catagonus wagneri*), indicating that they arose after the split between Suidae and the sister family Tayassuidae (SF3). These results underscore the importance of using primary sequence data, not only to broaden the range of species included in the analyses, but also to correctly ascertain whether the *PCSK9* gene structure and function have been preserved in a given species.

**Fig. 3:**
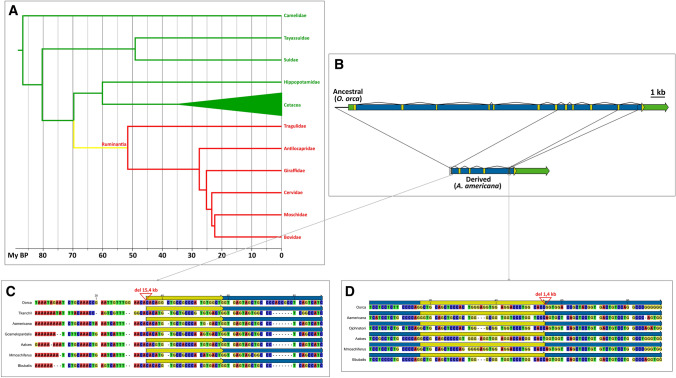
*PCSK9* pseudogenization in Cetartiodactyla. **a** Phylogenetic tree of Cetartiodactyla (adapted from (Hassanin et al. 2012)) indicating *PCSK9* gene status: green—functional; red—pseudogene; yellow—branch where pseudogenization occurred, at an undetermined time point. **b** *PCSK9* gene/pseudogene structure in Cetartiodactyla (yellow—coding sequence; green—non-coding exonic sequence; blue—intronic sequence). Lines connecting the ancestral (gene) and derived (pseudogene) representations indicate deletion breakpoints recognizable by sequence alignments. **c**, **d** Alignments of sequences flanking ancient deletions; arrowheads indicate points where intervening sequences in the *O. orca* genome were removed to facilitate the alignments

The second important result obtained from the analyses performed in Cetartiodactyla was the existence of a marked contrast in the status of *PCSK9* within the order. All evidence suggested that PCKS9 is functional in Camelidae, Suidae, Tayassuidae, Hippopotamidae and Cetacea: the gene had the typical 12-exon structure, with conservation of splice junctions and internal exon sizes (with the noted exception in Suidae), and there was also conservation of the catalytic triad and oxyanion hole residues, and the 25 cysteines in the catalytic and C-terminal domains (Fig. 3, SF4), as well as gene expression in the liver. On the contrary, Pecora had the remnants of a *PCSK9* pseudogene, including exons 8, 9, 10, 12 and parts of exons 7 and 11. Interestingly, two major structural alterations affecting 38 of the 39 species analysed could be reconstituted: a large deletion extending from the *BSND*-*PCSK9* intergenic region to exon 7, and a smaller deletion from exon 11 to intron 11 (Fig. 3, SF5). The first of these structural alterations was also recognizable in the lesser mouse-deer (*Tragulus kanchil*), in which a different deletion, extending from intron 8 to the *PCSK9*-*USP24* intragenic region, was identified in the genomic sequence. The loss of *PCSK9* in Cetartiodactyla can thus be inferred to have taken place in the time interval from the Whippomorpha-Ruminantia split (ca. 70 My bp) to the divergence of Ruminantia (ca. 52 My bp) (Hassanin et al. 2012).

**Fig. 4:**
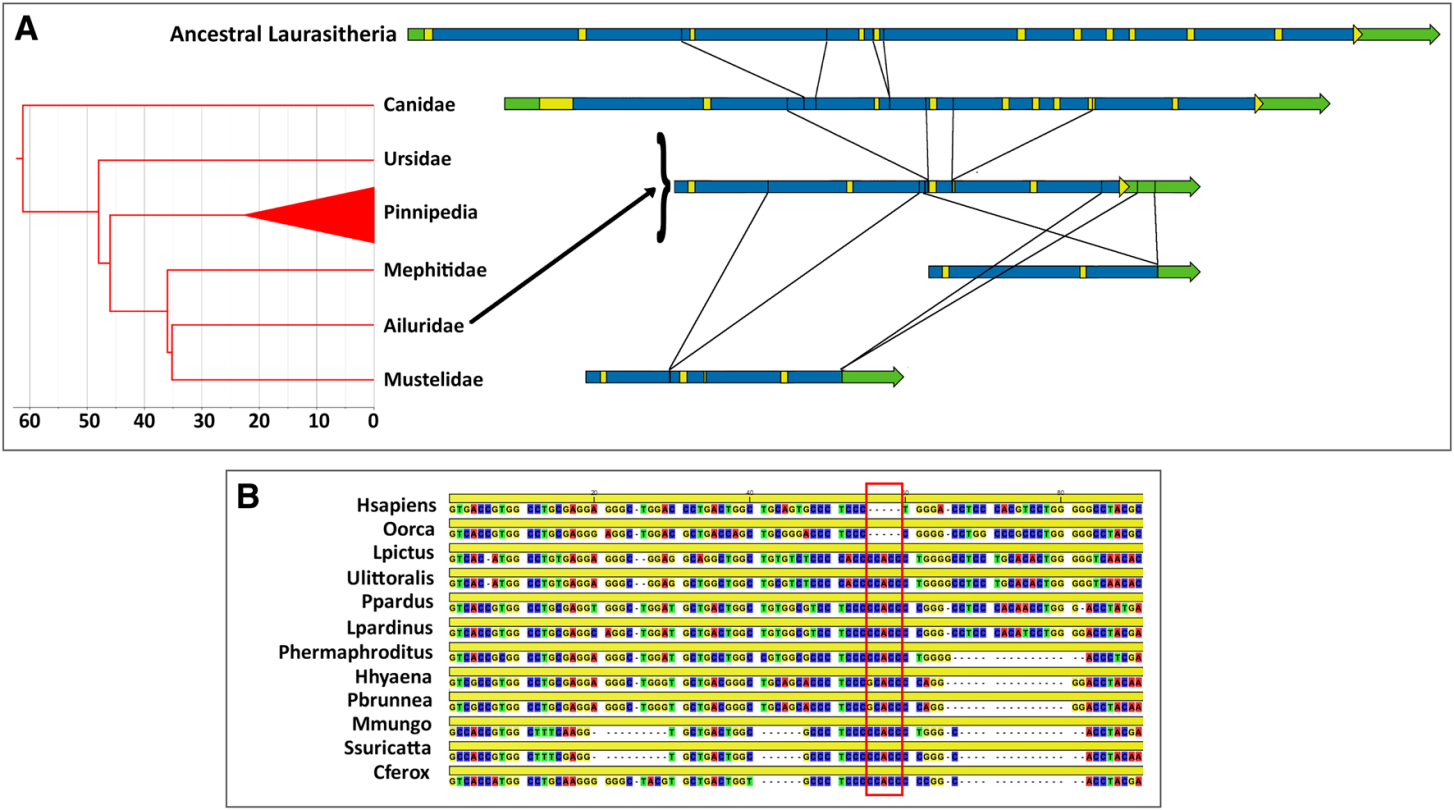
*PCSK9* pseudogenization in Carnivora. **a** Structures of the ancestral *PCSK9* gene in Laurasitheria and *PCSK9* pseudogenes found in different Caniformia (yellow—coding sequence; green—non-coding exonic sequence; blue—intronic sequence). The Caniformia tree was adapted from (Nyakatura and Bininda-Emonds 2012). Lines connecting gene or pseudogene representations indicate deletion breakpoints recognizable by sequence alignments. **b** Partial alignment of exon 12 sequences from *H. sapiens*, *O. orca*, two Canidae and eight Feliformia species

In Carnivora, none of the 63 species analysed had a functional gene, but there was a marked difference in the pattern of gene inactivation between the suborders Caniformia and Feliformia. In Caniformia, traces of gradual gene degradation over evolutionary time could still be found when comparing the basal Canidae to more recent families (Fig. 4). Indeed, while Canidae typically lack only exons 3 and 5, two additional deletions caused the loss of exons 4, 7 to 9 and part of exon 10 after the Ursidae-Canidae split, as evidenced by the sequences of Ursidae, Pinnipedia and Ailuridae. Finally, an additional two internal deletions led to the losses of exon 2 and the coding region of exon 12 seen in Mustelidae, while in the single species of Mephitidae available, a large deletion has left only exons 1, 2 and part of exon 12's non-coding region. The breakpoints involved in this deletion, as well as those for the two Mustelidae-specific deletions and the earlier deletion from intron 6 to exon 10, are still identifiable through sequence comparisons among the relevant species. In Feliformia, on the other hand, only exon 12 has been preserved in Felidae and Hyaenidae, while Viverridae also contained a fragment of exon 6, and Herpestidae and Eupleridae have retained exons 10 to 12 (ST2). Interestingly, all extant exon 12 sequences from Feliformia and Canidae (although not from other Caniformia) seemed to share a 5-bp insertion (Fig. 4), suggesting that *PCSK9* inactivation took place before the Caniformia-Feliformia split and therefore very early in the evolution of Carnivora.

**Fig. 5:**
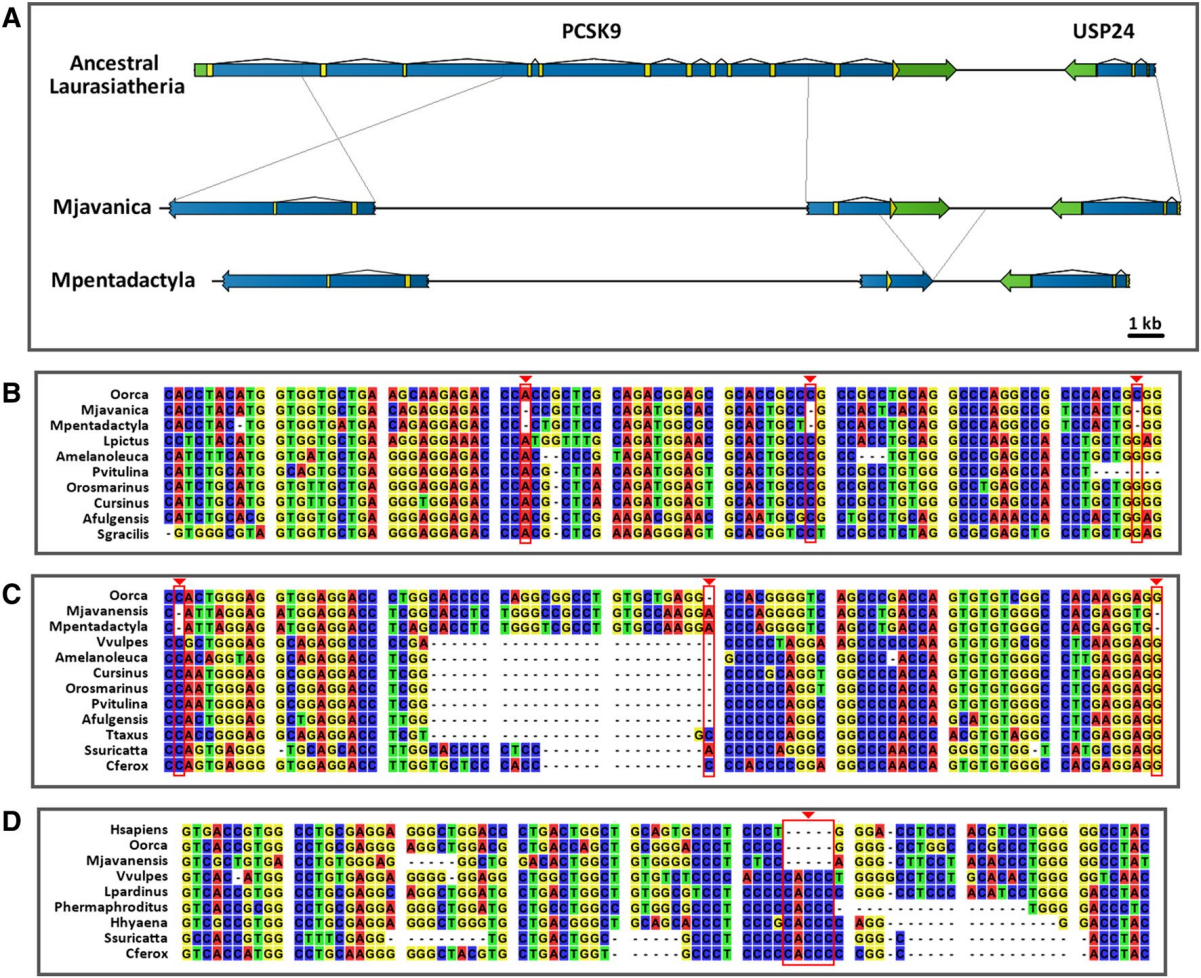
*PCSK9* pseudogenization in Pholidota. **a** Structure of the *PCSK9* locus (and part of the adjacent *USP24* locus) in ancestral Laurasiatheria and two pangolin species (yellow—coding sequence; green—non-coding exonic sequence; blue—intronic sequence). Lines connecting gene or pseudogene representations indicate deletion breakpoints recognizable by sequence alignments. **b**, **c**—None of the six frameshift mutations in exons 2 (**b**) or 11 (**c**) that are shared by pangolins (boxed and indicated with arrowhead) are present in Carnivora. **d** The 5-bp insertion found in Canidae and Feliformia (boxed and indicated with arrowhead) is not shared with pangolins

In Pholidota, which includes only one family comprised of eight species (Gaubert and Antunes 2005), *PCSK9* was inactivated in the two species for which there was available data (the Sunda pangolin, *Manis javanis*, and the Chinese pangolin, *Manis pentadactyla*). The inactivation of the gene occurred before the two species diverged, as attested by similar alterations in gene structure (losses of exons 1 and exons 4 to 10, and inversion affecting exons 2 and 3) and sequence (e.g. three frameshift mutations in exon 2) (ST2, Fig. 5). This similarity is not surprising, as the two species are closely related (Gaudin et al. 2009), but it should be noted that the sequences also provided evidence of additional recent gene degradation, as only the Chinese pangolin has lost exon 12 (Fig. 5). As Pholidota and Carnivora are thought to be sister orders (Murphy et al. 2001; Foley et al. 2016; Ronquist et al. 2016; Chen et al. 2017; Liu et al. 2017), it was interesting to investigate if the inactivation of *PCSK9* could have occurred before their divergence. However, no evidence in support of this hypothesis was found; indeed, the 5-bp insertion in exon 12 that seems to have occurred in Carnivora before the Caniformia-Feliformia split is not present in Pholidota and, conversely, none of the six frameshift mutations in exons 2 and 11 of both species of Pholidota occur in Carnivora (Fig. 5).

**Fig. 6:**
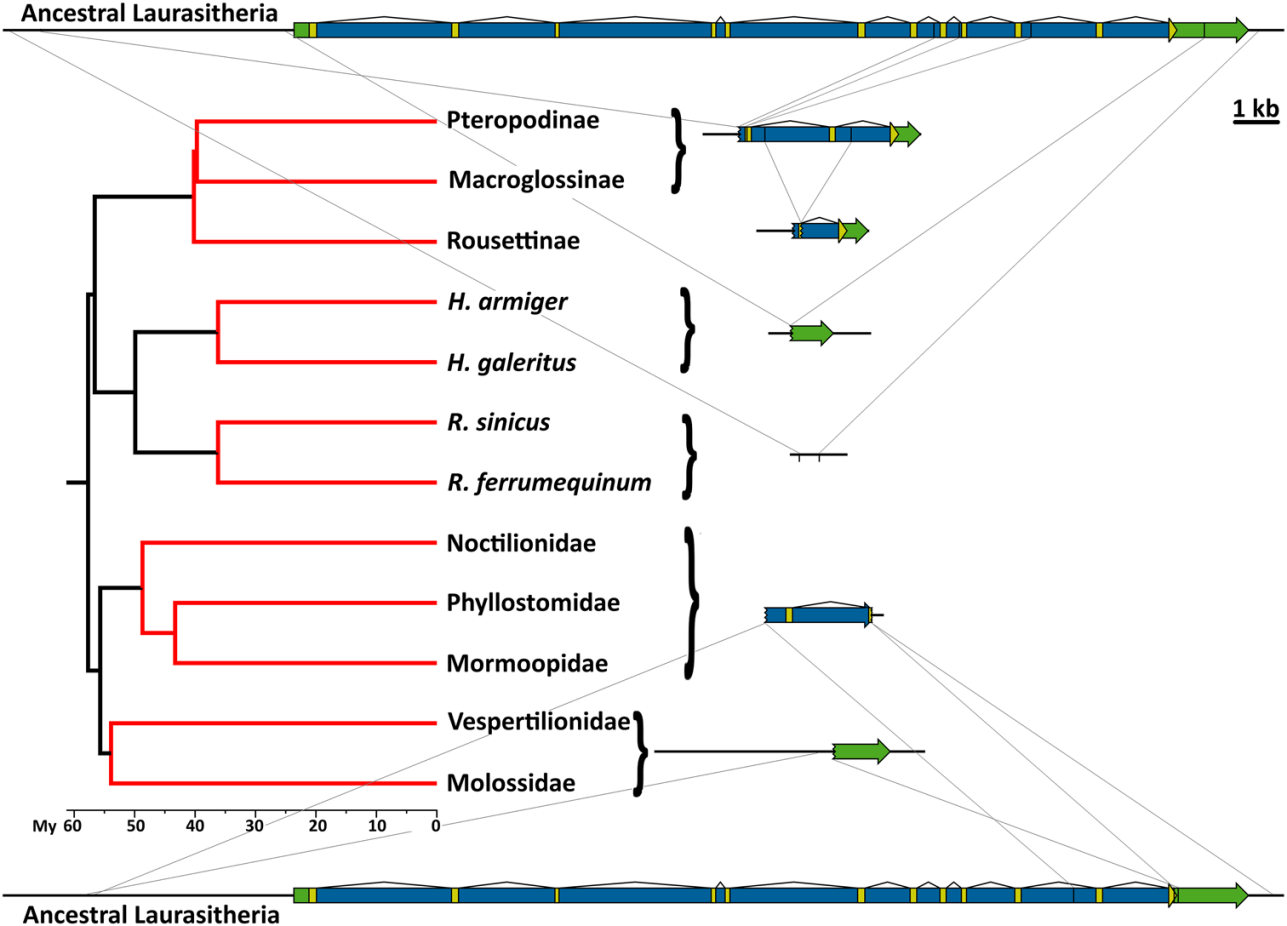
*PCSK9* pseudogenization in Chiroptera. Structures of the *PCSK9* locus in ancestral Laurasiatheria and Chiroptera (yellow—coding sequence; green—non-coding exonic sequence; blue—intronic sequence). The Chiroptera phylogenetic tree was adapted from (Shi and Rabosky 2015). Lines connecting gene or pseudogene representations indicate deletion breakpoints recognizable by sequence alignments. Note that in the two Rhinolophidae species no sequences homologous to *PCSK9* were found; homology was restricted to sequences flanking the gene

Chiroptera is another order in which *PCSK9* has apparently been inactivated in all species. Furthermore, observed levels of gene degradation were generally more extensive than in Carnivora, Pholidota or Ruminantia (Cetartiodactyla). No sequences homologous to the gene's coding region were preserved in 19 of the 37 species analysed, and the entire gene sequence (including the long 3′ UTR) was lost in two of them (ST2). This high level of gene degradation greatly complicates the identification of sequence or structural commonalities that could help illuminate the evolutionary history of *PCSK9* in Chiroptera. Indeed, among the eleven families represented, there were only two cases in which species from different families shared protein-inactivating alterations (Fig. 6): (a) structural defects, including an identical deletion of all the sequences corresponding to exon 12 after codon 671 (as well as an internal 30-bp deletion in exon 12) in Phyllostomidae, Mormoopidae and Noctilionidae suggest that *PCSK9* was inactivated before the latter's divergence, estimated at about 49 My bp (Shi and Rabosky 2015); and (b) a deletion extending from the *BSND*-*PCSK9* intergenic region to a few bases after *PCSK9*'s stop codon in Vespertilionidae and the Brazilian free-tailed bat (*Tadarida brasiliensis*) (Molossidae), which diverged ~ 54 My bp (Shi and Rabosky 2015). Nevertheless, intra-familial comparisons provided information on the patterns of *PCSK9* pseudogenization in bats and estimates of minimum ages of gene inactivation (Fig. 6). Among pteropodids, Pteroropodinae and Macroglossinae shared two deletions, one of them extending from the *BSND*-*PCSK9* intergenic region to intron 7, and the other eliminating exons 9 and 10. The first of these deletions was also recognizable in the Egyptian fruit bat (*Rousettus aegyptiacus*) (while the second was concealed by an additional deletion), indicating that pseudogenization in Pteropodidae occurred more than 40 My bp (Shi and Rabosky 2015). The great roundleaf bat and Cantor's roundleaf bat (*Hipposideros armiger* and *Hipposideros galeritus*) lost all but part of *PCSK9*'s 3'-UTR through an identical deletion, which therefore happened over 36 My bp. Finally, the Chinese rufous horseshoe bat and the greater horseshoe bat (*Rhinolophus sinicus* and *Rhinolophus ferrumequinum*) share an identical whole gene deletion, which can be dated to before 36 My bp (Shi and Rabosky 2015).

Perissodactyla is similar to Chiroptera, Pholidota and Carnivora in that *PCSK9* has clearly become a pseudogene in all species. However, there are important differences. First, exon structure was remarkably preserved, with only one case of exon loss among the 12 species analysed (ST2). Furthermore, although there were multiple protein-inactivating mutations (frameshift insertions and deletions as well as nonsense point mutations) in all species, and many of these were shared by all members of a family, none of the protein-inactivating mutations was present in all species. This raised the possibility that the inactivation of *PCSK9* took place independently in different families. Given that more than 80% of the gene sequence was preserved in all species, this hypothesis could be tested by comparing the intensity of selection at different points in the evolution of Perissodactyla to the intensity of selection in Cetacea—the largest Laurasiatheria clade where the gene's function has been preserved. As expected, selective pressure in Perissodactyla (as a whole) was relaxed relative to Cetacea, as detected using RELAX (Wertheim et al. 2015), which compares identical values of ω for both groups of branches and ω_Peri_ = ${\upomega }_{{{\text{Ceta}}}}^{{\text{K}}}$, where K is the selection intensity parameter (K = 0.05, p = 0.000). On the other hand, relaxation of selective pressure was *not* detected in the ancestral Perissodactyla branch relative to Cetacea, even though evidence for relaxation was found when comparing Perissodactyla's post-divergence branches to either Cetacea (K = 0.04, p = 0.000) or ancestral Perissodactyla (K = 0.05, p = 0.039). Taken together, these results suggest that *PCSK9* was still under significant selective pressure before the Hippomorpha-Ceratomorpha split, and that gene inactivation may have occurred independently in different Perissodactyla lineages.

**Fig. 7:**
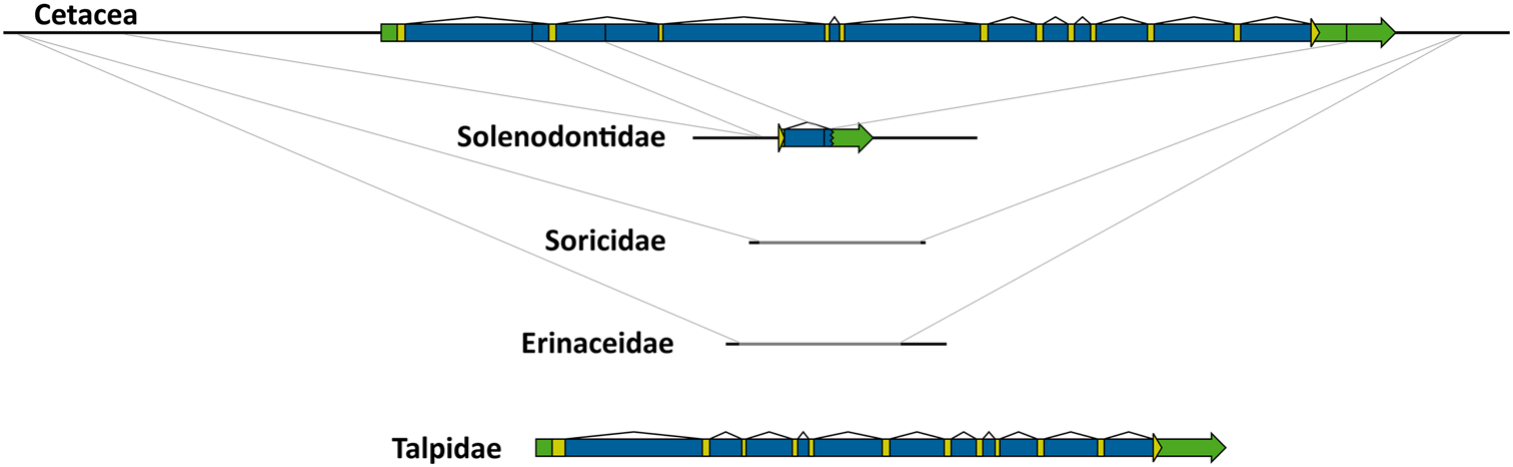
*PCSK9* gene status in Eulipotyphla. Schematic comparison of the *PCSK9* gene locus and flanking sequences in different families of Eulipotyphla and Cetacea (yellow—coding sequence; green—non-coding exonic sequence; blue—intronic sequence). Note that in Soricidae and Erinaceidae no sequences homologous to *PCSK9* were found; homology (black segments) was restricted to short intergenic regions (black segments) which, in these families, flanked sequences with no recognizable homology to *PCSK9* (gray segments)

Eulipotyphla also presented a unique *PCSK9* pattern: it was the order of Laurasiatheria that displayed the largest divergence among species, ranging from an apparently functional gene to the absence of recognizable gene remnants. In the star-nosed mole (*Condylura cristata*), the reference sequence lacked exons 2 and 3, but this large deletion was an error resulting from a sequence assembly gap that could be corrected using raw sequence data. The corrected sequence, like the two other Talpidae sequences available, suggested that *PCSK9* is still functional in these species, as it contains no frameshift mutations and encodes a protein with all the conserved cysteine, catalytic and oxyanion hole residues (SF6). There were some "unusual" features, such as the losses of 15 bp in exon 9 and 6 bp in exon 11, but these occurred in the three species, suggesting that they are ancestral in Talpidae and likely have no impact on protein function. At the other extreme were Soricidae (the common, Asian house and Indochinese shrews— *Sorex araneus*, *Suncus murinus* and *Crocidura indochinensis*, respectively) and the European hedgehog (*Erinaceus europaeus*) (Erinaceidae), where no gene remnants could be detected, either by BLAST searches against raw sequence data (even though the neighbouring *BSND* and *USP24* gene sequences could easily be recovered) or by homology searches against the available Soricidae *BSND*-*USP24* intergenic sequences using sequences from other Laurasiatheria species as probes (Fig. 7). Finally, an "intermediate” situation occurred in the Hispaniolan solenodon (*Solenodon paradoxus*) (Solenodontidae), where sequences corresponding to exon 2 and exon 12 (non-coding only) were brought close together through large deletions but could still be identified (Fig. 7, SF7). Given that Solenodontidae are thought to occupy the basal position among Eulipotyphla (Sato et al. 2016; Casewell et al. 2019), these results also suggested that *PCSK9* inactivation occurred independently in different lineages within the order.

### The *PCSK9* landscape in Euarchontoglires is marked by high conservation, with some exceptions

In Euarchontoglires, as in Laurasiatheria, the abundance of data allowed for broad analyses and the identification of general patterns. In Rodentia, *PCSK9* sequences were recovered and/or assembled from a total of 111 species (see “Materials and methods” for selection criteria). However, there were several cases in which deficient coverage precluded the recovery of complete coding sequences, mostly in species where exon capture or RNA-seq data from tissues other than the liver were used. Therefore, two species with coverage restricted to less than 40% of the coding sequences were excluded from the study, whereas species with coverage in the 80-98% range were used in some of the subsequent analyses.

The lesser Egyptian jerboa (*Jaculus jaculus*)*,* one of the species for which the data only yielded an incomplete coding sequence (< 30%) turned out to be interesting. Genomic coverage of the *PCSK9* locus was far from ideal as it contained numerous gaps, but even with these limitations it was intriguing that only three exons could be detected. Close inspection revealed the presence of frameshift deletions in exons 6 and 11, the latter of which encompassing Cys600 and Cys601 (residue numbering of the human protein), as well as independent missense mutations affecting Cys562, Cys588, and Cys654 (SF8). As all suspected alterations were confirmed using primary sequence data, it is likely that *PCSK9* has been inactivated in *J. jaculus*. Since this species was the sole member of the family Dipodidae for which genomic data was available, an attempt was made to amplify exonic sequences from another family member using degenerate exonic primers designed for pan-rodent amplification. The northern birch mouse was chosen as the test species because it belongs to Sminthinae, the most basal sub-family of Dipodidae (Pisano et al. 2015), while mouse, CHO cells and the great gerbil (see below) were used as controls. Amplification and sequencing of exons 6 and 11 showed that neither of the inactivating alterations found in the Egyptian jerboa was present in the birch mouse. The analysis was therefore extended to include all exons except 1, 10 and 12, thus yielding ~ 50% of the coding sequence (excluding primer binding sites), including two residues from the catalytic triad, the oxyanion hole residue, and 11 of the cysteines in the catalytic and the V-domain. Additionally, no protein-truncating alterations or mutations affecting key residues were detected, suggesting that *PCSK9* is functional in the birch mouse*,* and that it is not generally lost among dipodids (SF8).

The subsequent release of genomic data from two other dipodids—the Gobi jerboa (*Allactaga bullata*) and the meadow jumping mouse (*Zapus hudsonius*)—provided another opportunity to ascertain whether the pseudogenization of *PCSK9* was specific to the Egyptian jerboa or a more general feature within the family. In the Gobi jerboa, the closest relative of the Egyptian jerboa analysed, the whole coding sequence could be recovered through a combination of contig and raw sequence data searches. It presents three unique indels, but none of them are frame-shifting or affect catalytically important residues or conserved cysteines. On the other hand, two conserved cysteines are indeed altered by missense mutations: Cys562 (the same alteration as seen in the Egyptian jerboa) and Cys588 (a different alteration) (SF8), raising the possibility of gene inactivation in this species. As for the meadow jumping mouse, the species apparently lost exons 1, 2, 4 and 5, had four frame-shifting indels and an independent nonsense mutation in codon 419, as well as missense mutations affecting N317, S386, C562, C588 and C601 (SF8). None of the apparent defects could be confirmed by raw sequence data analysis as these are unavailable, but nevertheless it seems highly likely that *PCSK9* has also become a pseudogene in the jumping mouse. It is possible that such inactivation occurred in a common ancestor of Zapodinae, Allactaginae and Dipodinae, but given the absence of unquestionable inactivating mutations (its only guaranteed "suspicious" alteration is C562Y), it is also possible that PCSK9 was still functional in such an ancestor—and indeed in the Gobi jerboa, *A. bullata*. Supporting this notion, no relaxation of selective pressure was detected in the ancestral Dipodidae/*A. bullata* branch compared to the remaining Myomorpha. Additionally, C562Y was not present in Pedetidae, Rhyzomyidae or Spalacidae, but because codon 562 is in the "primer shadow" of exon 11, it is unclear whether the C562Y alteration is common to all dipodids or if it occurred after Sminthinae split from the remainder Dipodidae. Taken together, these results suggest that *PCSK9* was independently inactivated in the Egyptian jerboa and the jumping mouse, but the possibility remains that inactivation took place in a common ancestor—and, in the latter case, the question at which point in dipodid phylogeny it occurred remains open.

**Fig. 8:**
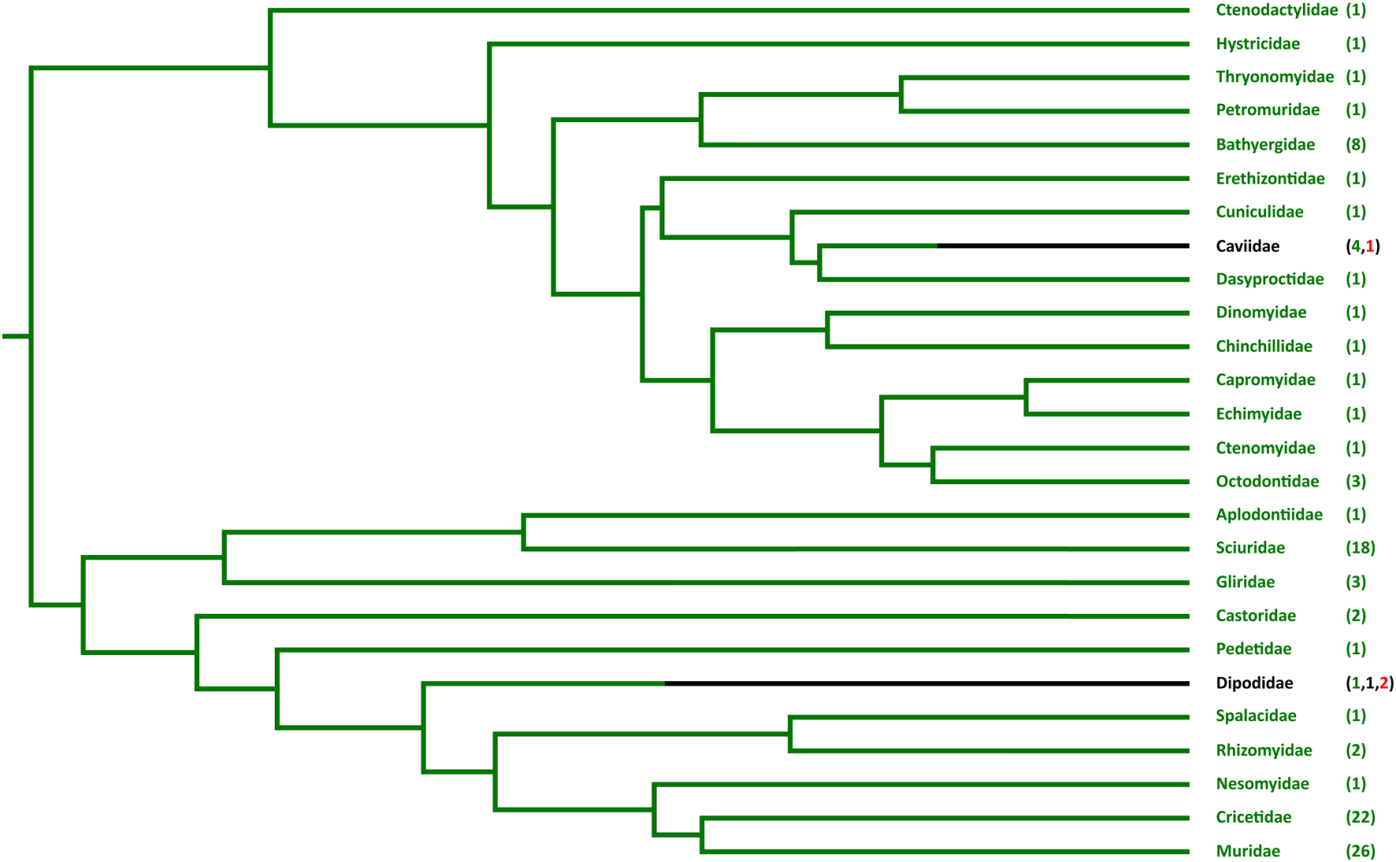
*PCSK9* gene landscape in rodents. Phylogenetic tree (adapted from (Fabre et al. 2012)) of rodent families included in this study and number of species retaining the gene (green) where pseudogenization has occurred (red) or where the status is unclear (black). Pseudogenization within a family is indicated by a shift from green to black in the family branch

Evidence from the remaining 105 rodent species (representing 24 families) nevertheless suggests that *PCSK9* pseudogenization is a rare event among rodents. Indeed, only one clear additional example was detected: the capybara (*Hydrochoerus hydrochaeris*), whose sequence contained a variety of nonsense and frameshift mutations (Fig. 8). No such mutations were found in any other species: the catalytic and oxanion residues were completely conserved, and the gene was invariably found to be expressed in species for which RNA data was analysed. Interestingly, more sequence variability—both in terms of exon size and amino acid sequence—was found among Rodentia than in other clades, such as Cetacea or Afrotheria. For example, apart from the "more typical" size variations in exons 1 and 12, in-frame insertions or deletions were also observed in exons 3, 4, 8, 9, 10 and 11. Three of these are particularly worth noting. First, there was a 12-bp extension at the 3′ end of exon 3 in several cricetids, but the frequency of this variant within the family is unclear. DNA data suggests that the ancestral cricetid sequence around the exon 3–intron 3 junction (also shared with species from other families, e.g. the house mouse, *Mus musculus*) is compatible with alternative splicing giving rise to both a "canonical” (short) form of exon 3 and a “long” form with an extra 12 bp (SF9). However, only the "canonical” form was found in RNA sequences from Neotominae (two species), only the “long” form was detected in Sigmodontinae (three species), while in Arvicolinae (two species) the “long” form was present in a small minority (~ 10%) of the transcripts. It is conceivable that some of these differences can be due to mutations affecting one or the other of the two possible splice donor sites. Indeed, two of the 17 cricetids for which DNA data was available had such alterations, leading to a long exon 3 in one case, and a short exon 3 in the other (SF9). However, they are unlikely to explain the majority of cases, and certainly do not explain the differences in splice-site utilization among species where both options are available. In keeping with database annotations and the results from species for which both RNA and DNA sequences were available, cricetid DNA sequences were annotated as giving rise to the short version of exon 3, except in the cases where mutation has made the long form obligatory (the golden hamster, *Mesocricetus auratus*), or RNA data indicated otherwise (the hispid cotton rat, *Sigmodon hispidus*). Second, length variations were found in exon 4, which are interesting in that they were close to the 5′ end and therefore structurally similar to the exon 3 extension found in Cricetidae and to the exon 4 extension found in Suidae. Finally, two 3-bp deletions, one in exon 10 and the other in exon 11, were found in all Ctenohystrica species, suggesting that they are ancestral in the clade.

At the amino acid sequence level, the catalytic domain was highly conserved in rodents, as observed in Cetacea and Afrotheria. This high level of conservation was not limited to the residues critically involved in catalytic activity (as mentioned above), but extended to the six cysteines involved in disulfide bonds (100% conserved), and even the GTRFHR peptide found to be disordered in the human protein, which has 97–100% of conserved residues. However, average identity levels within the catalytic domain were lower in Rodentia (88%) than in Cetacea (98%) or Afrotheria (90%). This difference was more pronounced in the V-domain, where average identity in rodents dropped to 76%, compared to 97% in Cetacea, and 85% in Afrotheria. Furthermore, three of the 18 V-domain cysteines typically present in PCSK9 were not completely conserved in rodents: besides the case of the Gobi jerboa (see above), Cys608 was altered in the greater cane rat (*Thryonomys swinderianus*) Cys562 in the Djungarian hamster (*Phodopus sungorus*—Cricetinae) and Cys588 was mutated in three different species. No other mutations with potentially deleterious effects on protein function were detected in any of these species. Furthermore, two of the species with alterations affecting Cys588 belong to the subfamily Gerbillinae and both continue to express *PCSK9* in the kidney, while the third belongs to the closely related Deomyinae, suggesting that the relevant cysteine was replaced before the separation of the two subfamilies. Additional support for this notion came from direct amplification and sequencing of a third member of the family Gerbillinae, the great gerbil, which also had a missense mutation at codon 588. As the two subfamilies show no evidence of reduced selective pressure as compared to the remaining rodent species, are thought to have diverged more than 23 My bp and no truncating mutations or any other alterations affecting key residues were found in any of the four species, *PCSK9* seems to be still functional in Gerbillinae and Deomyinae.

Besides rodents and primates (see below), the Euarchontoglires clade also comprises the orders Dermoptera, Scandentia and Lagomorpha. Among the ten species belonging to these orders for which sequences could be analysed, two reference sequences had to be corrected: the Sunda flying lemur (*Galeopterus variegatus*) because it encoded a Cys635Arg substitution that was ruled out by raw sequence data analysis—which also revealed three missense alterations in the reference sequence; and the european rabbit (*Oryctolagus cuniculus*) because of a sequence gap encompassing the junction between intron 2 and exon 3, which had also caused annotation errors. Furthermore, RNA data from the rabbit revealed the presence of a 15-bp 5′ extension in exon 4 relative to the ancestral Euarchontoglires form of exon 4 (SF10). The same extension was also found in the RNA sequences available for other Leporidae, and resulted from alterations in intron 3, apparently common to all Leporidae, that created a new splice acceptor site upstream of the ancestral site (SF10). All species of Dermoptera, Scandentia and Lagomorpha had apparently normal *PCSK9* genes, except for the American pika (*Ochotona princeps*). *PCSK9* has clearly undergone pseudogenization in this species, as several exons were lost, and the remaining had three nonsense mutations, all of which were confirmed by raw sequence data. At present, and in the absence of additional data, it is not possible to determine whether the inactivation is common to the entire family Ochotonidae, or a restricted phenomenon.

**Fig. 9:**
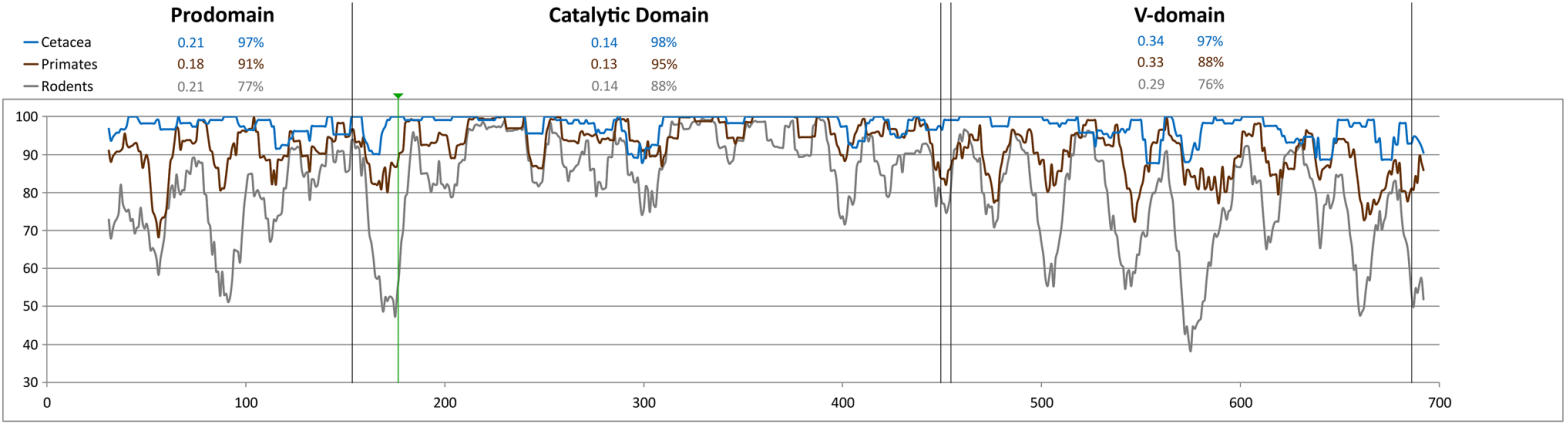
Amino acid conservation and selective pressure (ω) in Cetacea, primates and rodents. Pairwise identity values for Cetacea (blue), primates (brown) and rodents (gray) are shown (graph) for residues outside the signal peptide (31 to 692 in the human protein numbering). Limits of the prodomain, catalytic and C-terminal V-domain are marked by black vertical lines. The position of the codon spanning the exon 3–exon 4 junction is indicated by a green line and arrowhead. Average identity values (as percentages) and values of ω are given for each of the three domains

In primates, only partial coding sequences could be recovered for five species due to incomplete coverage. Missing sequences ranged from less than 20 bp to more than 700 bp, precluding the use of three sequences in some analyses. Regardless of these limitations, evidence pointed to the existence of a functional gene in all 86 species, with normal intron–exon junctions, complete conservation of the four catalytically crucial residues and the 24 structurally important cysteines, as well as gene expression in all species for which RNA sequence data was available. In fact, complete conservation was seen in 56% of residues outside the signal peptide, and this percentage rose to 69% in the catalytic domain. Average identity values in the catalytic (95%) and V-domain (88%) were clearly higher than in rodents, although not as high as in Cetacea (Fig. 9). It would be tempting to attribute the different levels of amino acid conservation observed in primates, rodents and cetaceans to differences in life-history traits, e.g. the much shorter generation time of rodents. However, lower levels of sequence conservation could also result from a relaxation of selective pressure on *PCSK9*. To distinguish between these two possibilities, the ratios of the rates of non-synonymous and synonymous substitutions (ω) between primates, rodents and cetaceans were compared. No evidence of reduced selective pressure was detected in rodents or primates compared to cetaceans, either when considering the whole mature protein, or each functional domain separately. On the other hand, the intensity of selective pressure was found to differ among functional domains, with ω values increasing to more than double from 0.13–0.14 in the catalytic domain to 0.29–0.34 in the V-domain (Fig. 9).

At the structural level, *PCSK9* was also more conserved in primates than in rodents: length variability was almost exclusively restricted to the regions coding for the signal peptide and the extreme C-terminus (after the end of the V-domain), with only three exceptions. Interestingly, signal peptide length variation was observed not only among species but in several cases also within a species, as has previously been described in humans (Chen et al. 2005). Close to the end of the coding sequence, the three *Saguinus* species shared a termination codon at the position corresponding to amino acid 689 in humans, the two *Plecturocebus* had a termination codon at position 686, while the Philippine tarsier (*Tarsius syrichta*) lacked residue 684. As in Afrotheria, it seems unlikely that these alterations have a functional impact on the protein, particularly when considering that: (a) the time divergences for *Saguinus* and *Plecturocebus* species are estimated to be older than 7 My and 4 My, respectively (Springer et al. 2019); (b) no evidence of relaxed selective pressure was found in these species compared to the remaining primates. Taken together, Euarchontoglires provided a picture of general strong negative selective pressure leading to high levels of *PCSK9* conservation within the clade, in stark contrast to the pattern found in Laurasiatheria.

## Discussion

The primary goal of this study was to obtain a general picture of the patterns, tempo and mechanisms of *PCSK9* pseudogenization in eutherian mammals. However, the results are also relevant for two other (and more general) aspects of molecular phylogenetic studies. The first relates to the high level of limitations and errors detected in sequences and/or annotations retrieved from public databases, including reference sequences, a problem other authors have also encountered (Turakhia et al. 2020; Sharma and Hiller 2020). For example, the sequencing gaps in the rabbit and the star-nosed mole genomes can lead to errors in evolutionary studies by suggesting gene loss events that did not take place. Sequencing gaps can also cause the inclusion of intronic sequences in the gene's coding region as a result of the annotation process, thus influencing the results of phylogenetic analyses, as previously noted (Springer et al. 2019). The same type of mistakes can also take place due to faulty assignment of a start codon, as apparently has occurred in *PCSK9* in some species of Primata and Sciuridae. Interestingly, using the most recent reference sequences was not a guarantee to avoid these problems. Indeed, in sporadic cases it can compound them, as illustrated by the reference sequences of the domestic pig and the common bottlenose dolphin, for which the current versions (as of December 31st, 2019) contain errors that were not present in previous ones. These results illustrate the current lag between the generation of genomic data and its thorough curation, as well as the consequent importance of using primary sequence data (e.g. SRA) to ensure that better supported conclusions can be drawn from phylogenetic studies.

The second type of result of potentially broad relevance for phylogenetic analyses can be illustrated by the gradual *PCSK9* gene degradation observed in Caniformia. As mentioned above, Ursidae, Pinnipedia and Ailuridae shared two intragenic deletions absent in Canidae. Thus, in order for Canidae and Ailuridae to be sister families, as suggested in a previous study (Agnarsson et al. 2010), the same deletions had to arise independently in two different lineages, which is highly unlikely. And although the position of Ailuridae within the Carnivora tree is not considered controversial, this example shows how the reconstruction of "gene decay" can be used to resolve this type of controversy, should it arise. More generally, one can point to the various examples in which a combination of established phylogeny and common genetic alterations was used to date *PCSK9* gene inactivation, as cases where the common losses could be used to recover uncertain phylogenies. In other words, decaying genes represent a good and unexplored source of the type of rare and *irreversible* genetic alterations that can be instrumental for resolving difficult phylogenies.

Studying the patterns of pseudogenization of a gene requires criteria to decide whether the gene is functional or not. This task is simple in cases of exon-scale deletions, as well as frameshift or nonsense mutations leading to *early* protein truncations. And since PCSK9 mutant proteins lacking catalytic activity are not secreted, and therefore unable to regulate LDLR turnover rates (Seidah et al. 2003; Maxwell et al. 2005), the same can be said of missense mutations affecting the residues essential for PCSK9's auto-catalytic activity (the catalytic triad plus N317). But what about in-frame deletions, missense mutations affecting highly conserved residues, or late truncations? Evolutionary data, as well as available structural and functional information on the human protein were used to interpret these. For example, the extreme C-terminus is one of the least conserved regions of PCSK9 (Fig. 9), all residues after 683 are de-structured in the human protein (Piper et al. 2007), and truncating it at residue 681 or later has no functional impact (Zhao et al. 2006), suggesting that the same applies to all the late truncations identified. Additional support for this notion comes from the occurrence of late truncations in multiple lineages, without any evidence of accumulation of loss-of-function mutations, as well as from comparisons of the level of selective pressure between primate species with truncation at codons 684/686 and other primates—which did not support the possibility of selection relaxation in the former. In-frame indels were found at highest frequency in the N-terminal signal peptide, but given the broad requirements for its function as well its high degree of variability in humans and other species, these alterations are very likely functionally neutral. Within the catalytic domain, most amino acid insertions or deletions were due to either 3′ extensions in exon 3, 5′ extensions in exon 4, or in-frame indels in the beginning of exon 4. Therefore, they fall in one of the less conserved regions of the entire mature protein (Fig. 9), which includes one of the catalytic domain's two unstructured loops (Piper et al. 2007). A similar bias was observed within the pro-domain, with the majority of indels mapping either to the disordered 31–60 residue stretch or to the loop between strand β1 and helix α1 (Cunningham et al. 2007), both of which display lower levels of conservation than the rest of the pro-domain. Finally, in the V-domain, the majority of indels also map to disordered regions, either in the extreme C-terminus, or in the region corresponding to β4 in sub-domain M2 (Cunningham et al. 2007). The concentration of in-frame indels in regions where structural and conservation data indicate that alterations are likely to be tolerated suggests these have no impact on protein function. Three additional lines of evidence support this notion. First, many of the indels are shared by species which diverged a long time ago without evidence of gene degradation, as seen in the extreme example of the 3 bp deletions in exons 10 and 11 common to Ctenohystrica. In contrast, the Laurasiatheria lineages affected by pseudogenization have a widespread accumulation of inactivating alterations. Second, gene expression and therefore promoter function are also invariably conserved in species displaying in-frame indels. Finally, indels, like missense mutations, were more frequently observed in rodents than in cetaceans or primates. As previously mentioned, in the case of missense mutations, the available evidence suggested that this difference could be due to life history traits. In other words, rodents probe the mutational space faster than other groups—and this should apply similarly to indels and to point mutations. Taken together, these observations justified the option to not consider indels, by themselves, as evidence of gene inactivation, even when they were present in a single species. Support for this option also came from Talpidae. Indeed, two in-frame deletions that had been identified, at an early stage of this work, as restricted to *Condylura cristata,* were later (with the availability of additional Talpidae sequences) found to be common to Uropsilinae and Scalopinae, and therefore must have occurred more than 35 My bp (Sato et al. 2016).

A similar decision on gene inactivation was made with regards to missense mutations affecting highly conserved residues (other than those involved in auto-catalysis), as many of the arguments presented above (e.g., higher frequency of variants among rodents due to more efficient probing of mutation space, conservation of expression, and absence of gene degradation over extended periods of time) are also valid in such cases. Indeed, as mentioned above, such arguments apply to the Cys588 alterations found in Gerbillinae and Deomyinae. Furthermore, Cys588 forms a bridge with Cys562 (Cunningham et al. 2007), which was altered in a cricetid species, suggesting that alterations in both residues are tolerated. If this is the case, then the common ancestor of Zapodinae, Allactaginae and Dipodinae might have had a functional *PCSK9*, a notion also supported by the absence of relaxation of selective pressure. On the other hand, two of its descendants have lost *PCSK9* function, raising the possibility that such losses represent an example of degradation of a gene that encodes a defective protein. Finally, it is also possible that the losses of Cys562 and Cys588 are tolerated in some species and not in others, a question that could be answered with additional data from Gerbillinae, Deomyinae and Dipodidae. This possibility is also relevant in the case of the greater cane rate, which carries a C608F alteration. Cys608 forms a bridge with Cys679 (Cunningham et al. 2007), and truncation of the human protein at residue 679 (or 680, but not 681) causes loss of function due to protein retention in the ER (Zhao et al. 2006); therefore, it is possible that PCSK9 is also inactive in the greater cane rat. However, it should be pointed out that, although both C679X and C679S human mutant PCSK9 are not secreted, and are consequently non-functional, C679A is partially secreted [18], so it is conceivable that some alterations in residue 608 are also tolerated.

The above considerations suggest that among the species considered in this study, there could be some unidentified cases in which *PCSK9* has become non-functional. However, such cases are probably rare and do not significantly alter the general patterns of pseudogenization discerned. So, the answer to the question of whether *PCSK9* has become evolutionarily unimportant is clearly no—in Euarchontoglires. If the gene had become irrelevant, one would expect a pattern of frequent losses randomly distributed, affecting large clades as well as individual species, and this is not what was observed. It is true that *PCSK9* was lost in the American pika (or perhaps in all Ochotonidae) and in a few rodent species, but it should be noted there are other examples of genes that can hardly be considered evolutionarily unimportant which have been lost more than once in mammals—e.g. *MMP20*, causing loss of enamel in baleen whales and sloths (Meredith et al. 2011a), or *GULO*, whose pseudogenization led to inability to synthesize vitamin C in several mammalian lineages (Yang 2013). More generally, the growing availability of genomic data has led to the identification of multiple cases of species or group-specific gene loss, to a renewed interest in gene loss as evolutionary mechanism and to an increased awareness that even deleterious gene losses have some probability of becoming fixed during evolution (Albalat and Cañestro 2016). Despite some notable exceptions, the general evolutionary pattern of *PCSK9* in Euarchontoglires is one of widespread conservation, driven by strong negative selective pressure. This is particularly evident in primates where, contrary to earlier suggestions (Ding et al. 2007), not even a single "suspicious" case was detected among 86 species. Thus, there remains little doubt that *PCSK9* was under selective pressure in the ancestral lineages leading up to our species. Whether that pressure is still acting on modern humans is an open question, particularly in light of the occurrence of null alleles in some human populations, apparently without adverse health effects (Zhao et al. 2006; Hooper et al. 2007). Loss of PCSK9 function leads to a decrease in circulating LDL-cholesterol levels, which might have been problematic for our ancestors who probably had a cholesterol-poor diet, but can be particularly advantageous for people on cholesterol-rich diets—such as those common in many industrialized countries. In this respect, it is interesting that null alleles of human *PCSK9* have originated in Africa, and have been argued to display evidence of positive selection (Mbikay et al. 2007; Sirois et al. 2008). An intriguing—and admittedly highly speculative—hypothesis to explain this data would be that those alleles arose or were selected for in pastoralist societies relying mostly on cattle products (including blood) as a source of nutrition, as loss of *PCSK9* might have a protective effect against such a "hypercholesterolemic" diet.

**Fig. 10:**
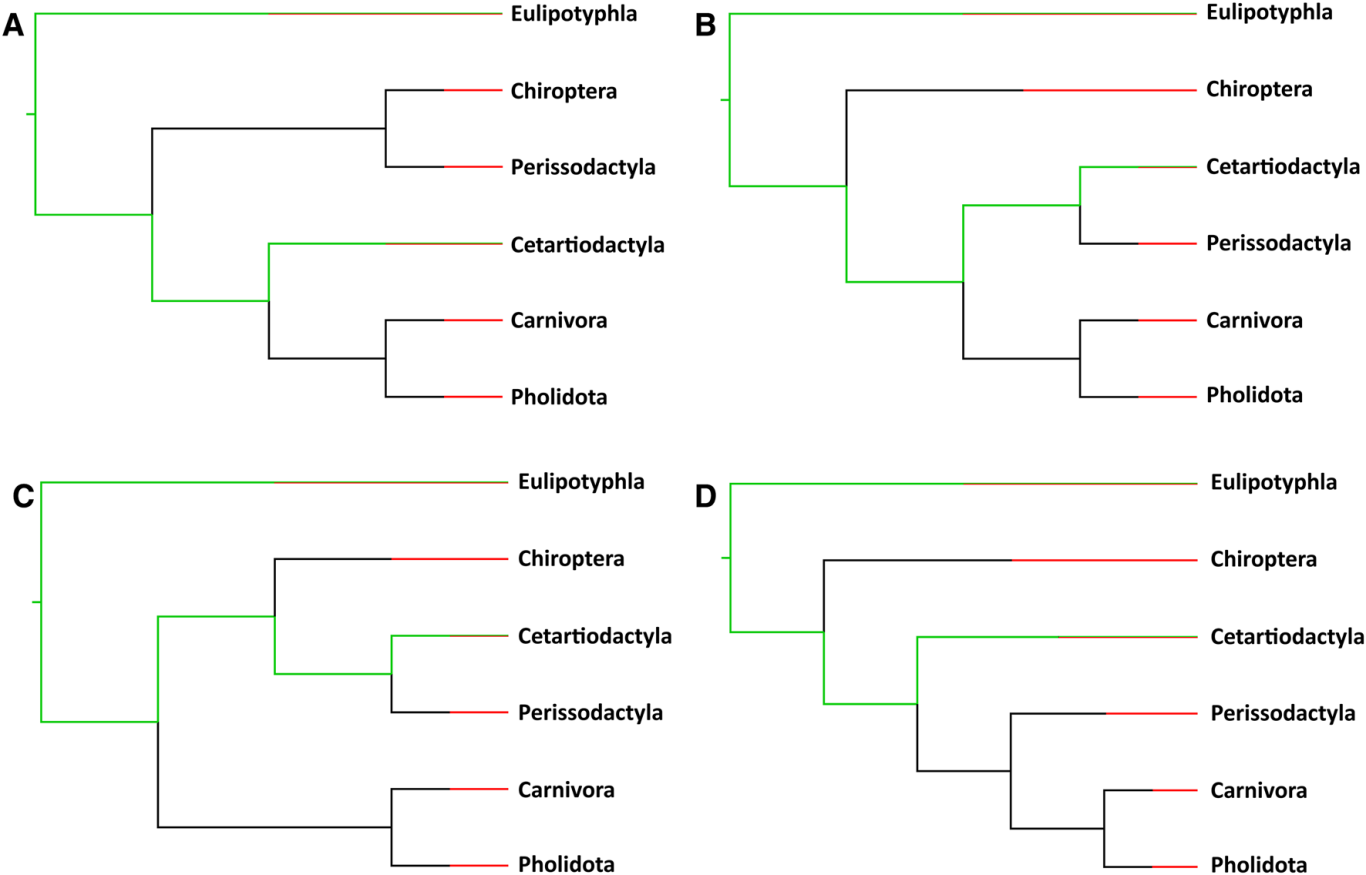
Different Laurasiatheria phylogenetic trees proposed recently. Trees (displaying only Laurasiatheria orders) are adapted from: **a** Chen et al. (2017), **b** Liu et al. (2017), **c** Ronquist et al. (2016), **d** Foley et al. (2016). Green indicates a functional *PCSK9* gene, red a pseudogene and black uncertainty regarding gene status. Note the co-existence (after an arbitrarily set time point) of separate lineages with a gene or a pseudogene in both Eulipotyphla and Cetartiodactyla

The pattern is radically different in Laurasitheria, a clade where the loss of *PCSK9* is widespread. Interestingly, this is *not* the ancestral state in Laurasiatheria, and pseudogenization occurred not only once, but multiple times and independently in different lineages. How many times exactly it has occurred depends, among other factors, on order placement in the phylogeny of Laurasiatheria, a hotly and sometimes acrimoniously debated issue—with at least four different trees advocated recently (Fig. 10) (Foley et al. 2016; Ronquist et al. 2016; Chen et al. 2017; Liu et al. 2017). However, there is general agreement on a basal position for Eulipotyphla which, since Talpidae have retained *PCSK9*, implies at least one independent loss event in Eulipotyphla. Likewise, pseudogenization had to happen independently in Ruminantia, implying that at least three independent gene loss events took place in Laurasiatheria. But there is no evidence for a common loss in different orders, not even for Carnivora and Pholidota, whose sister relationship is generally accepted. Furthermore, although the data are consistent with a single event leading to pseudogenization in each of these orders, the situation is less clear in bats. Indeed, given the minimum ages of datable gene inactivations and the general high level of *PCSK9* degradation, it is tempting to speculate that pseudogenization in Chiroptera happened early the order's history, and likely only once, but it remains formally possible that it occurred independently in different bat clades. In Perissodactyla, the data seem to indicate that each of the three families lost the gene independently. And finally at least two losses occurred in Eulipotyphla if Solenodontidae is the basal family, as several studies (Meredith et al. 2011b; Sato et al. 2016; Casewell et al. 2019; Springer et al. 2019) (but not all (O’Leary et al. 2013)) indicate, and three losses if Talpidae and Soricidae are sister families, as has been proposed (Meredith et al. 2011b). Therefore, a total of seven to nine independent pseudogenization events seems a more likely estimate.

Irrespective of the exact number of gene loss events, three features stand out in the pattern of *PCSK9* pseudogenization in Laurasiatheria. First, loss of gene function affects most species, but it was not the clade's ancestral state. Second, in each of the clades affected by a specific loss (be it an order, suborder or family), that loss seems to have occurred early in the clade's evolution. Third, in clades not affected by losses, the gene is still under negative selective pressure. What kind of model could explain such patterns? The fact that losses occur repeatedly suggests that ancestral Laurasitheria, although having a functional *PCSK9*, had undergone some genetically determined alteration in cholesterol metabolism which made them more susceptible to losing the gene. But the observed pattern is not consistent with the widespread, random losses expected to occur if the gene had become unimportant: *PCSK9* was not only preserved in certain lineages (which could simply be due to chance), but in those lineages there are no examples of pseudogenization, and the gene is under considerable selective pressure. In fact, selective pressure in Cetacea, the Laurasiatheria clade for which more data is available, is similar to that in rodents or primates, which do not exhibit susceptibility to *PCSK9* loss. A possible solution to this apparent paradox lies in the timing of pseudogenization events. If, as the data suggest, gene losses occurred early in the evolution of groups when body plans and physiology were "being established", the profound alterations such processes entail (with all its evolutionary trials and errors), may have provided the opportunity for additional genetic alterations leading to changes in cholesterol metabolism which, in combination with the pre-existing one, completed the process of rendering *PCSK9* dispensable. On the contrary, in the lineages where such additional alterations did not occur, *PCSK9* continued to be selected for once the physiology was "stabilized". This model, which is similar to the one proposed to explain patterns of enamel and tooth loss among mammals (Davit-Béal et al. 2009; Meredith et al. 2011a), thus implies that PCSK9 pseudogenization in Laurasiatheria might be an example of gene loss due to evolution. It also suggests two areas to explore in the future, as more genomic data become available: first, to what extent the dichotomy of early loss / long-term retention holds true—particularly in the case of Talpidae, for which data is still limited; and second, what was the—presumably genetic—event that made Laurasiatheria more susceptible to *PCSK9* loss. Perhaps more importantly, and independently of whether or not this model is correct, the data presented here strongly suggests a fundamental (ancestral) difference in the regulation of cholesterol metabolism between Euarchontoglires and Laurasiatheria, and this has important implications for the use of Laurasiatheria species (e.g. pigs) as animal models of human cholesterol-related diseases.

## Supplementary information

Below is the link to the electronic supplementary material.

Electronic supplementary material 1 (PDF 15481 kb)

Electronic supplementary material 2 (PDF 840 kb)

Electronic supplementary material 3 (XLS 70 kb)

Electronic supplementary material 4 (XLSX 39 kb)

Electronic supplementary material 5 (XLSX 11 kb)

Electronic supplementary material 6 (FA 543 kb)

Electronic supplementary material 7 (PDF 596 kb)

Electronic supplementary material 8 (PDF 957 kb)

Electronic supplementary material 9 (PDF 7164 kb)

Electronic supplementary material 10 (PDF 6507 kb)

Electronic supplementary material 11 (PDF 3684 kb)

Electronic supplementary material 12 (PDF 3278 kb)

Electronic supplementary material 13 (PDF 3053 kb)

Electronic supplementary material 14 (PDF 1901 kb)
