# Supplementary material for: Patterns and tempo of PCSK9 pseudogenizations suggest an ancient divergence in mammalian cholesterol homeostasis mechanisms
Source: Genetica. 2021 Jan 30;149(1):1–19. doi: 10.1007/s10709-021-00113-x (PMC7929951; doi:10.1007/s10709-021-00113-x)
Supplement: Supplementary file 1 — Electronic supplementary material 1 (PDF 15481 kb) [file 10709_2021_113_MOESM1_ESM.pdf]

Supplemental Figure 1.

Alignments of DNA coding sequences (A) and protein sequences (B) of Afrotheria and Xenarthra species *vs H. sapiens*. "Corrected" sequences (i.e. disregarding the 1 bp deletion in exon 12 and the 10 bp extension of exon11) were used for *D. novemcinctus*

A

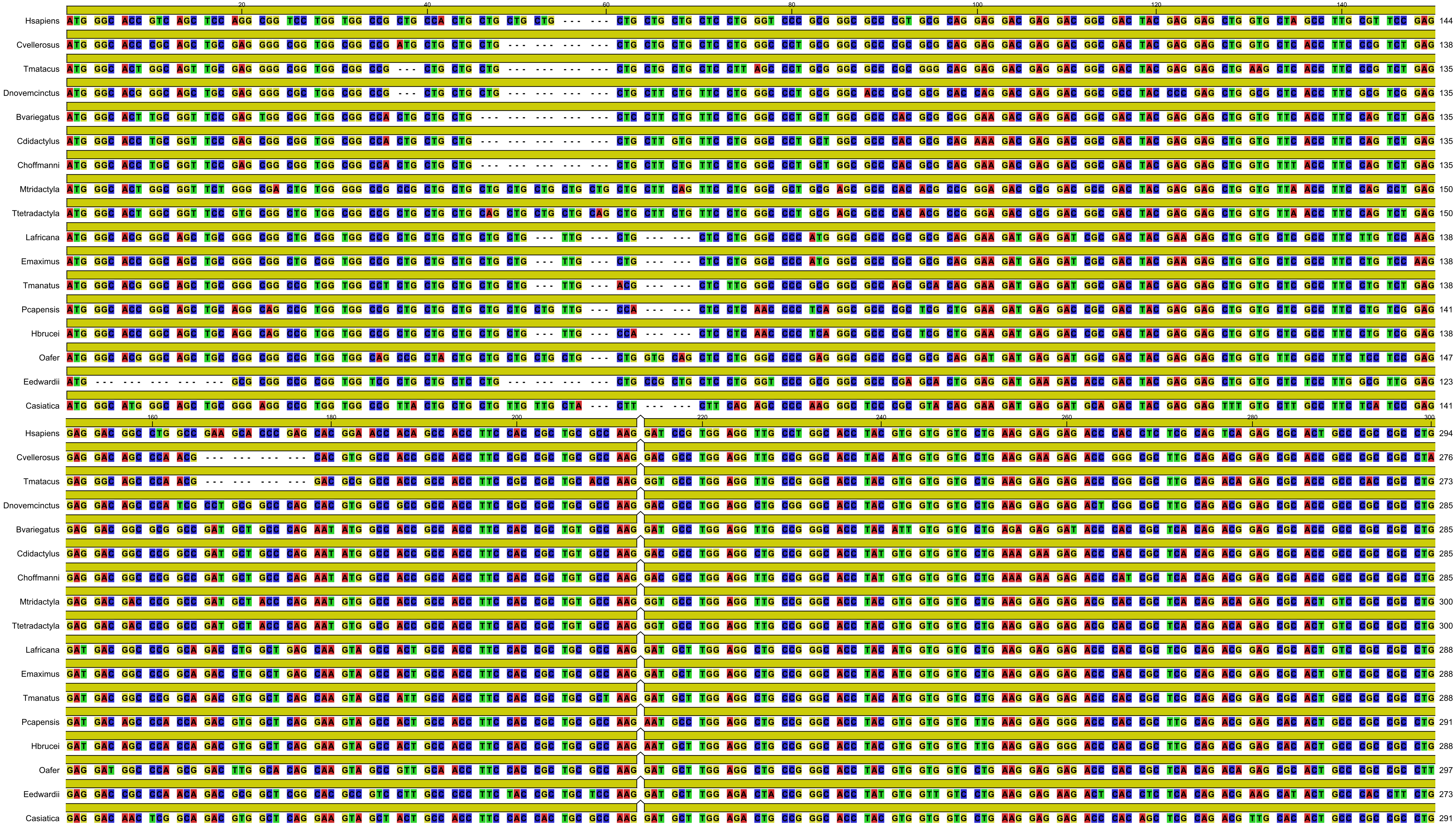

```
              320        340        360        380        400        420        440
Hsapiens      CAG GCC CAG GCT GCC CGC CGG GGA TAC CTC ACC AAG ATC CTG CAT GTC TTC CAT GGC CTT CTT CCT GGC TTC CTG GTG AAG ATG AGT GGC GAC CTG CTG GAG CTG GCC TTG AAG TTG CCC CAT GTC GAC TAC ATC GAG GAG GAC TCC TCT 444
Cvellerosus   CAG ACC CAG GCC GCC CGC CGG GGC TAC CTC ACC AAG ATA CTG CAC GTC TTC CAT GGC CTC TTC CCT GGC TTC TTG GTG AAG ATG AGT GGC GAT CTG CTG GAC CTG GCC CTG AGG TTG CCC CAC GTG GAG TAT ATC GAG GAA GAC TCC TCT 426
Tmatacus      CAG ACC TGG GCC GCC CGC CGA GGC TAC CTC ACC AAG ATC CTG CAC ATC TTC CAT GGC CTC TTC CCT GGC TTC TTG GTG AAG ATG AGT GGC GAT CTG CTG GAC CTG GCC CTG AGG TTG CCC CAT GTG GAG TAT ATC GAG GAG GAC TCC TCT 423
Dnovemcinctus CAG ACC CGG GCC GCC CGC CAG GGC TAC CTC GCC GAG ATC CTG CAC GTC TTC CAC GAG CTC TTC CCC GGC TTC TTG GTG AAG ATG AGT GGC GAT CTG CTG GAC CTG GCC CTG AGG CTG CCC CAC GTG GAG TAC ATC GAG GAG GAC TCC TCT 435
Bvariegatus   CAG GCC CGC GCC GCC CGC CGG GGC TAC CTC ACC AAG ATC CTG CTC GTC TTC CAC GAC CTC TTC CCT GGC TTC CTG GTG AAG ATG AGC AGC GAC CTG CTG GAC CTG GCC CTG AGG CTG CCC CAC GTG GAG TAC ATT GAA GAA GAC TCC TCT 435
Cdidactylus   CAG GCC CGC GCC GCC CGC CGG GGC TAC CTC ACC AAG ATC CTG CAC GTC TTC CAC AAC CTC TTC CCT GGC TTC CTG GTG AAG ATG AGC AGC GAC CTC TTG GAC CTG GCC CTG TGG CTG CCC CAC GTG GAG TAC ATC GAG GAA GAC TCC TCT 435
Choffmanni    CAG GCC CGC GCT GCC CGC CGG GGC TAC CTC ACC AAG ATC CTG CAC GTC TTC CAC AAC CTC TTC CCT GGC TTC CTG GTG AAG ATG AGC AGC GAC CTC TTG GAC CTG GCC CTG CGG CTG CCC CAC GTG GAG TAC ATC GAG GAA GAC TCC TCT 435
Mtridactyla   CAG GCT CGC GCC GCC CGC CGG GGC TAC CTC ACC AAG ATC CTG CAT GTC TTC TAC GAC CTC TTC CCT GGC TTC CTG GTG AAG ATG AGC AGT GAC CTG CTG GAC CTG GCC CTG AGG CTG CCC CAC GTG GAG TAC ATC GAG GAA GAC TCC TCT 450
Ttetradactyla CAG GCT CGC GCC GCC CGC CGG GGC TAC CTC ACC AAG ATC CTG CAT GTC TTC TAT GAC CTC TTC CCT GGC TTC CTG GTG AAG ATG AGC AGC GAC CTG CTG GAC CTG GCC CTG AGG CTG CCC CAC GTG GAG TAC ATC GAG GAA GAC TCC TCT 450
Lafricana     CAG GCC CGT GCA GCC CGC CGG GGC TAC CTC ACG AAG ATT TTG CAT GTC TTT CAC GAT CTC TTC CCT GGC TTC CTG GTG AAG ATG AGT GGT GAC CTA CTG GAC CTG GCT CTG AGG TTG CCC CAC GTC GAG TAC GTC GAG GAA GAC TCC TCT 438
Emaximus      CAG GCC CGT GCA GCC CGC CGG GGC TAC CTC ACG AAG ATT TTG CAT GTC TTT CAC GAT CTC TTC CCT GGC TTC CTG GTG AAG ATG AGT GGT GAC CTA CTG GAC CTG GCT CTG AGG TTG CCC CAC GTC GAG TAC ATC GAG GAA GAC TCC TCT 438
Tmanatus      CAG TCC CGT GCT GCC CGC CGG GGC TAT CTC ACC AAG ATC CTG CAC GTC TTC CAC CGT CTC TTC CCT GGC TTC CTG GTG AAG ATG AGT GGC GAC CTG CTG GAC CTG GCC CTG AGG TTG CCC CAC GTC GAG TAC ATC GAG GAA GAC TCC TCT 438
Pcapensis     CAA TCC CGA GCG GCC CGC CGG GGC TAC CTC ACC AAA ATC CTG CGT ATC TTC CAT GAA CTC TTC CCT GGC TTC CTG GTG AAG ATG AGC GGT GAC CTA CTG GAT CTG GCC CTG AGG CTG CCC CAT GTT GAG TAC ATA GAG GAA GAC TCC TCT 441
Hbrucei       CAA TCC CGA GCG GCC CGC CGG GGC TAC CTC ACC AAA ATC CTG CGT ATC TTC CAC GAA CTC TTC CCT GGC TTC CTG GTG AAG ATG AGC GGT GAC CTA CTG GAT CTG GCC CTG AGG CTG CCC CAT GTT GAG TAC ATA GAG GAA GAC TCC TCT 438
Oafer         CAG TCC CGT GCT GCC CGC CGG GGT TAC CTC ACC AAG ATC CTG CAT GTC TTC CAC AAT CTC TTC CCT GGC TTC CTG GTG AAG ATG AGT GGC GAC CTG CTA GAC CTG GCC CTG AGG TTG CCC CAT GTT GAG TAC ATT GAG GAA GAC TCC TCT 447
Eedwardii     CAG TCC CGT GCT GCC CAC CGG GGC TAC CTC ACC AAA ATC CTG CAC ATC TTC CAC GAT CTC TTC CCC GGC TTT GTA GTG AGG ATG AGT GGT GAC CTG CTG GAC CTG GCC CTG CGG CTG CCC CAC GTT GAA TAC ATT GAG GAA GAC TCC TTC 423
Casiatica     CAG TCC CGC GCT GCC CGC CGG GGC TAC CTC ATC AAG ATC CTG CAC ATC TTC CAT GAT CTC TTT CCC GGC TTC CTG GTG AAG ATG AGT GGT GAC CTG CTG GAC CTG GCC CTG AGG CTG CCC CAC GTG GAA TAC ATC GAG GAA GAC TCC TCT 441
              460        480        500        520        540        560        580        600
Hsapiens      GTC TTT GCC CAG AGC ATC CCG TGG AAC CTG GAG CGG ATT ACC CCT CCA CGG TAC CGG GCG GAT GAA TAC CAG CCC CCC GAC GGA GGC AGC CTG GTG GAG GTG TAT CTC CTA GAC ACC AGC ATA CAG AGT GAC CAC CGG GAA ATC GAG GGC 594
Cvellerosus   GTC TTT GCC CAG AGC ATC CCC TGG AAC CTG GAG CGG ATC ATC CCT GTG GGA TAC CAA GCG GAT GGA CAC CAG CCC CGC AAC GGA GGC AGC CTG GTG GAG GTG TAT CTC TTA GAC ACC AGC GTC CAG AGC AGC CAC CGG GAA ATT GAG GGC 576
Tmatacus      GTC TTT GCC CAG AGC ATC CCC TGG AAC CTG GAG CGG ATC ATC CCT GTG GGA TAC CAA GTG GAC GGA CAG CAG CCC CCC AAC GGA GGC AGC CTG GTG GAG GTG TAT CTC TTA GAC ACC AGC GTC CAG AGC GGC CAC CGG GAA ATT GAG GGC 573
Dnovemcinctus GTC TTT GCC CAG AGC ATC CCC TGG AAC CTG GAG CGG ATC ATC CGC GTG GGA TAC CAA GCG AAC GGA CAC CAG CCC CCC AAT GCG GGC AGC CTG GTG GAG GTG CAT CTC CTG GAC ACC AGC GTC CAG AGT GGC CAC CGG GAA ATT GAG GGC 585
Bvariegatus   GTC TTT GCC CAG AGC ATC CCC TGG AAC CTC GGG CGG ATC ATC CCT GCG CAG TAC CAG GAG GAT GGA TAC CAG CCC CCC AGT GGA GGC GGC CTG GTG GAG GTC TAC CTC TTG GAC ACC AGC ATC CAG AGC AGC CAC CGG GAA ATC GAG GGC 585
Cdidactylus   GTC TTT GCC CAG AGC ATC CCC TGG AAC CTG GGG CGG ATC ATC CCT GCG CGG TAC CAG GAG GAT GGA TAC CAC CCC CCC ANN NNN NNN NNN NNN NNN NNN NNN NNN NNN NNN NNN NNN NNN NNN NNN NNN NNN NNN NNN NNN NNN NNN NNN 585
Choffmanni    GTC TTT GCC CAG AGC ATC CCC TGG AAC CTG GGG CGG ATC ATC CCT GCG CGG TAC CAG GAG GAT GGA TAC CAC CCC CCC AGT GGA GGC GGC CTG GTG GAG GTG TAC CTC TTG GAC ACC AGC ATC CAG AGT GGC CAC CGG GAA ATC GAG GGC 585
Mtridactyla   GTC TTT GCC CAG AGC ATC CCC TGG AAC CTG GCG CGG ATC CTG CCT GCG CGG TAC CAG GTG GAC GGA TAC CGA CCC CCT AAT GGA GGT GGC CTG GTG GAA GTG TGC CTC TTA GAC ACC AGC ATT CAG AGC GGT CAT CAG GAA ATC GAG GGC 600
Ttetradactyla GTC TTT GCC CAG AGC ATC CCC TGG AAC CTG GCG CGG ATC CTG CCT GCG CGG TAC CAG GTG GAC GGA TAC CGA CCC CCC AAC GGA GGT GGC CTG GTG GAA GTG TGC CTT TTA GAC ACC AGC ATT CAG AGC GGT CAT CAG GAA ATC GAG GGC 600
Lafricana     GTC TTT GCC CAG AGC ATC CCA TGG AAC CTG GAG CGG ATG ATC CCT GCG CAG TAC AAG GCG GAT GAC TAC CAG CCC CCC AAT GGC AGC GGC CTG GTG GAG GTA TAT CTC TTA GAC ACT AGC ATC CAG AGT GGC CAC CGG GAA ATT GAG GGC 588
Emaximus      GTC TTT GCC CAG AGC ATC CCA TGG AAC CTG GAG CGG ATG ATC CCT GCG CAG TAC AAG GCG GAT GAC TAC CAG CCC CCC AAT GGC AGC GGC CTG GTG GAG GTA TAT CTC TTA GAC ACT AGC ATC CAG AGT GGC CAC CGG GAA ATT GAG GGC 588
Tmanatus      GTC TTT GCC CAG AGC ATC CCG TGG AAC CTG GAG CGG ATT ATC CCT GCG CGT TAC AAG GCG AAC GAC TAC CAG CCC CCC AAT GGT GGC GGC CTG GTG GAG GTA TAT CTC TTA GAC ACC AGC ATC CAG AGT GGC CAC CGG GAA ATT GAG GGC 588
Pcapensis     GTC TTT GCC CAG AGC GTC CCG TGG AAC CTG GAG CGG ATT TTC CCT GCA CGA TAC AAG GCG GAT GAC TAC CAG CCC CCT AAT GGC GGT AGC CTG GTA GAG GTA TAT CTC TTA GAC ACC AGC ATC CAG AGT GGC CAC CGG GAA ATT GAG GGC 591
Hbrucei       GTC TTT GCC CAG AGC GTC CCG TGG AAC CTG GAG CGG ATT TTC CCT GCA CGA TAC AAG GCG GAT GAC TAC CAG CCC CCT AAT GGC GGT AGC CTG GTA GAG GTA TAT CTC TTA GAC ACC AGC ATC CAG AGT GGC CAC CGG GAA ATT GAG GGC 588
Oafer         GTC TTT GCC CAG AGC ATC CCA TGG AAC CTG GAG CGG ATT ATC CCT CCA CGG TAC AAA GCA GAT GAC TAC CAG CCT CAA AAT AGC AGC AGC CTC GTG GAG GTA TAC CTC TTA GAC ACC AGC ATC CAG AGT GGC CAC CGG GAA ATT GAG GGC 597
Eedwardii     GTT TTT GCT CAG AGC ATC CCA TGG AAC TTA GCG CGG ATC AAC CCT GCT CAG CAC AAG CTG GAT GAC CAC CTA CAC TCC AAC GAT AGC AGC ATC GTG GAG GTC TAT CTC TTA GAC ACC AGC ATC CAG AGT GGC CAC CGA GAA ATT GAA GGC 573
Casiatica     GTA TTT GCC CAG AGC ATC CCG TGG AAC CTG GAT CGG ATT ATC CCT GCT TGG TAC AAG GCA GAT GAC TTC CAA CCA CCC AAT GGT GGT GGC CTG GTG GAG GTG TAT CTC TTA GAC TCC AGC ATC CAG AGT GAC CAC CGG GAA TTT GAA GGG 591
```

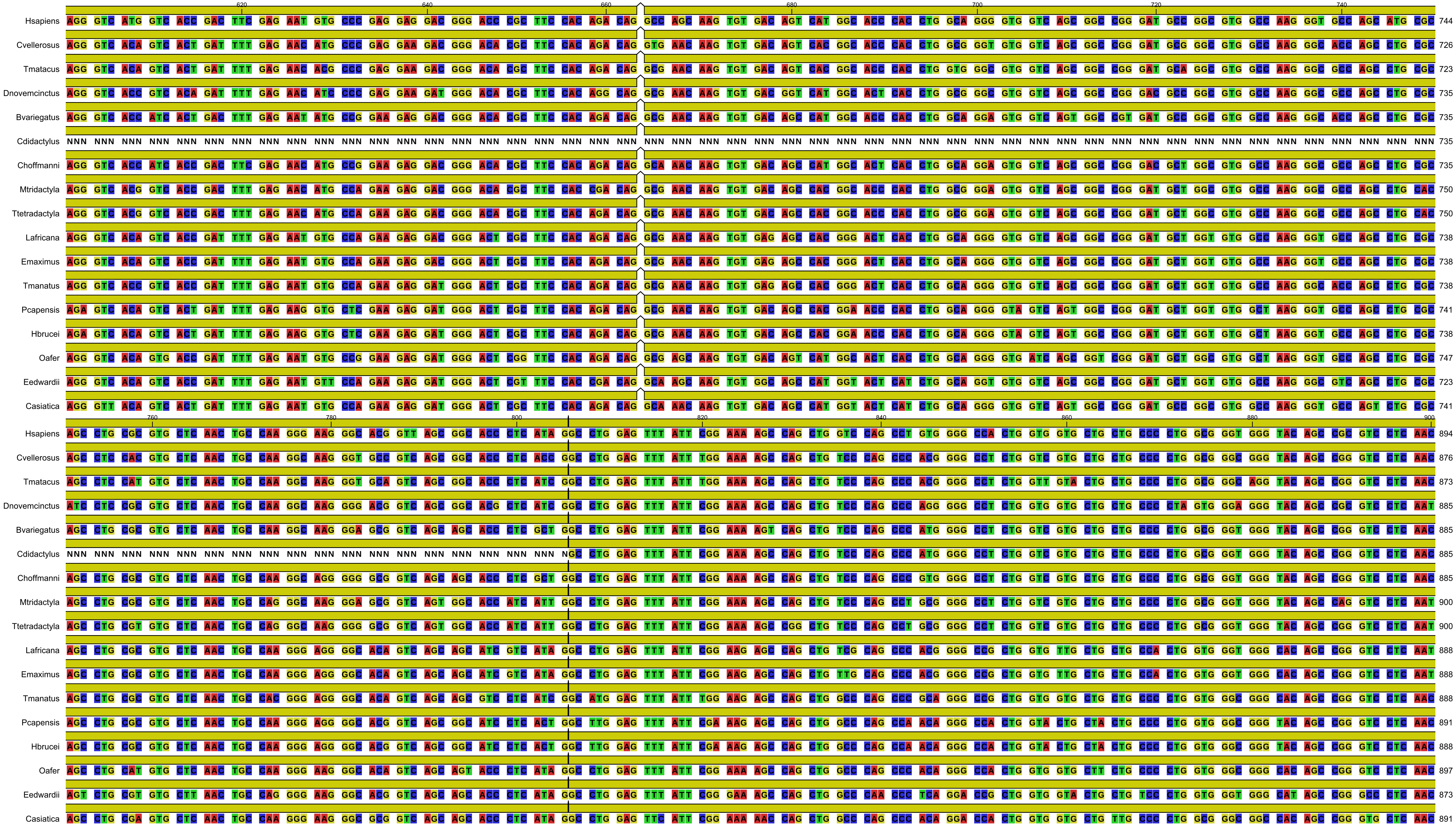
620
640
660
680
700
720
740
Hsapiens 744
Cvellerosus 726
Tmatacus 723
Dnovemcinctus 735
Bvariegatus 735
Cdidactylus 735
Choffmanni 735
Mtridactyla 750
Ttetradactyla 750
Lafricana 738
Emaximus 738
Tmanatus 738
Pcapensis 741
Hbrucei 738
Oafer 747
Eedwardii 723
Casiatica 741
760
780
800
820
840
860
880
900
Hsapiens 894
Cvellerosus 876
Tmatacus 873
Dnovemcinctus 885
Bvariegatus 885
Cdidactylus 885
Choffmanni 885
Mtridactyla 900
Ttetradactyla 900
Lafricana 888
Emaximus 888
Tmanatus 888
Pcapensis 891
Hbrucei 888
Oafer 897
Eedwardii 873
Casiatica 891

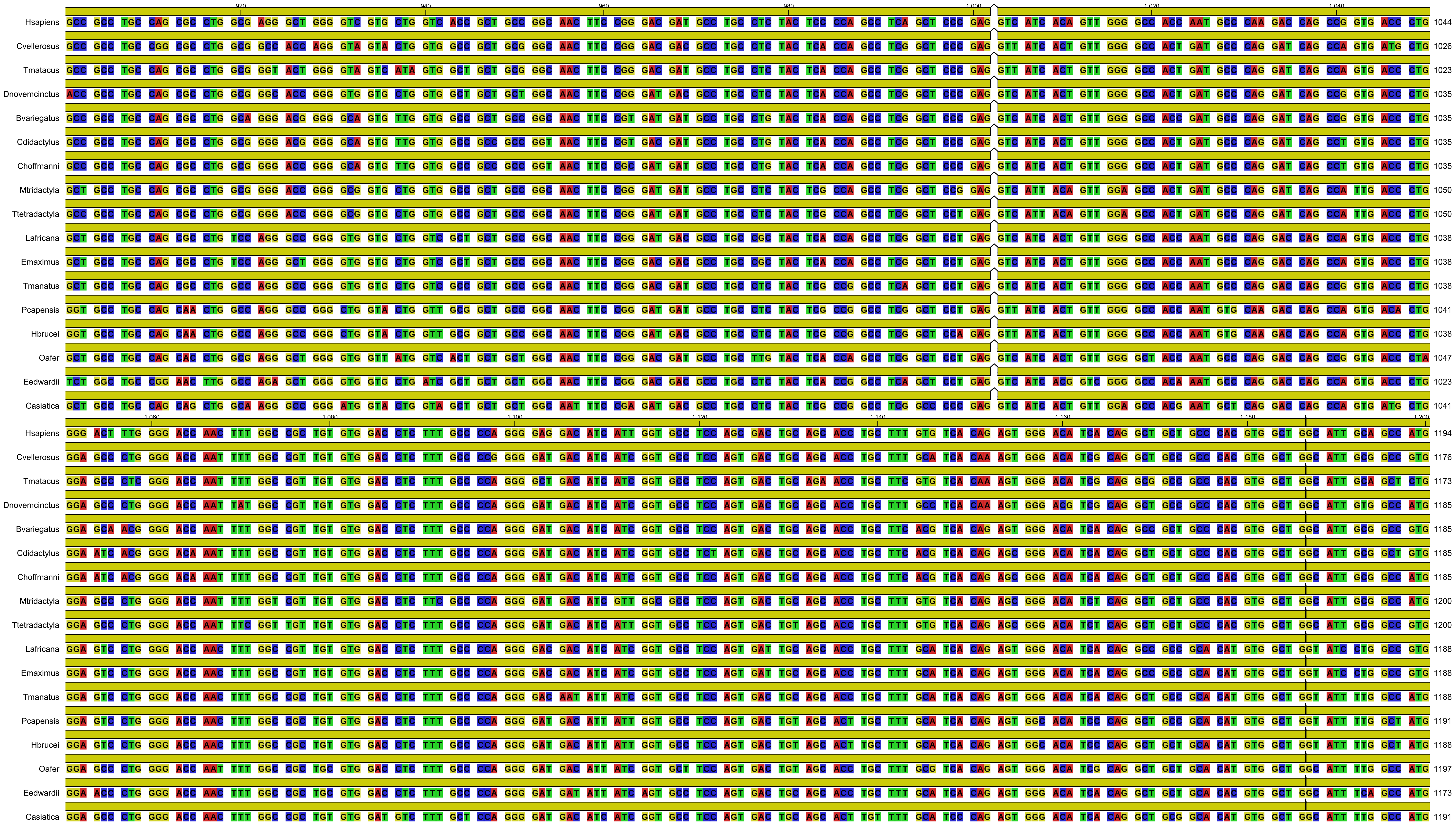
Hsapiens 1044
Cvellerosus 1026
Tmatacus 1023
Dnovemcinctus 1035
Bvariegatus 1035
Cdidactylus 1035
Choffmanni 1035
Mtridactyla 1050
Ttetradactyla 1050
Lafricana 1038
Emaximus 1038
Tmanatus 1038
Pcapensis 1041
Hbrucei 1038
Oafer 1047
Eedwardii 1023
Casiatica 1041
Hsapiens 1194
Cvellerosus 1176
Tmatacus 1173
Dnovemcinctus 1185
Bvariegatus 1185
Cdidactylus 1185
Choffmanni 1185
Mtridactyla 1200
Ttetradactyla 1200
Lafricana 1188
Emaximus 1188
Tmanatus 1188
Pcapensis 1191
Hbrucei 1188
Oafer 1197
Eedwardii 1173
Casiatica 1191

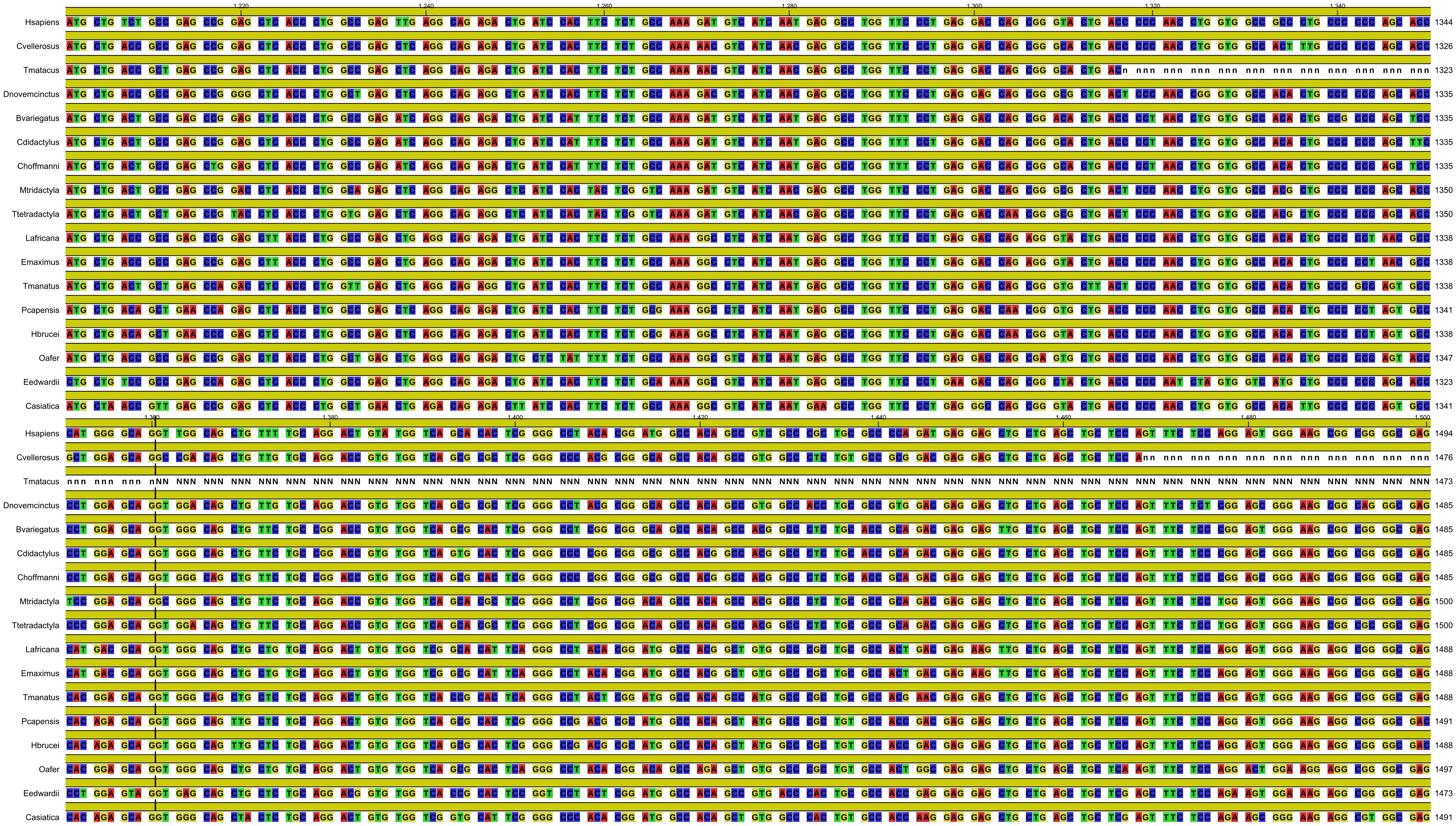
1,220 1,240 1,260 1,280 1,300 1,320 1,340
Hsapiens 1344
Cvellerosus 1326
Tmatacus 1323
Dnovemcinctus 1335
Bvariegatus 1335
Cdidactylus 1335
Choffmanni 1335
Mtridactyla 1350
Ttetradactyla 1350
Lafricana 1338
Emaximus 1338
Tmanatus 1338
Pcapensis 1341
Hbrucei 1338
Oafer 1347
Eedwardii 1323
Casiatica 1341
1,380 1,400 1,420 1,440 1,460 1,480 1,500
Hsapiens 1494
Cvellerosus 1476
Tmatacus 1473
Dnovemcinctus 1485
Bvariegatus 1485
Cdidactylus 1485
Choffmanni 1485
Mtridactyla 1500
Ttetradactyla 1500
Lafricana 1488
Emaximus 1488
Tmanatus 1488
Pcapensis 1491
Hbrucei 1488
Oafer 1497
Eedwardii 1473
Casiatica 1491

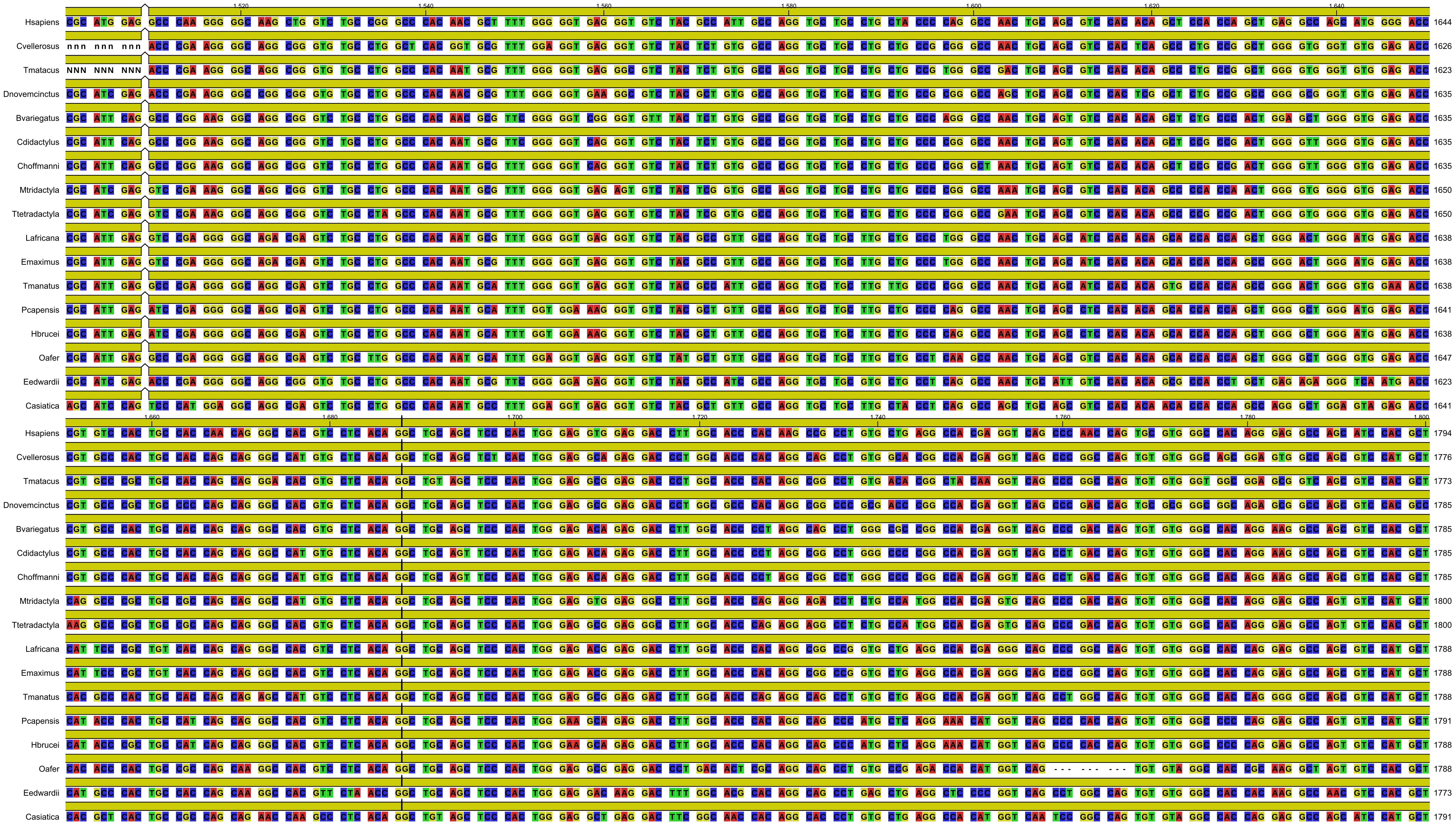
Hsapiens 1644
Cvellerosus 1626
Tmatacus 1623
Dnovemcinctus 1635
Bvariegatus 1635
Cdidactylus 1635
Choffmanni 1635
Mtridactyla 1650
Ttetradactyla 1650
Lafricana 1638
Emaximus 1638
Tmanatus 1638
Pcapensis 1641
Hbrucei 1638
Oafer 1647
Eedwardii 1623
Casiatica 1641
Hsapiens 1794
Cvellerosus 1776
Tmatacus 1773
Dnovemcinctus 1785
Bvariegatus 1785
Cdidactylus 1785
Choffmanni 1785
Mtridactyla 1800
Ttetradactyla 1800
Lafricana 1788
Emaximus 1788
Tmanatus 1788
Pcapensis 1791
Hbrucei 1788
Oafer 1788
Eedwardii 1773
Casiatica 1791

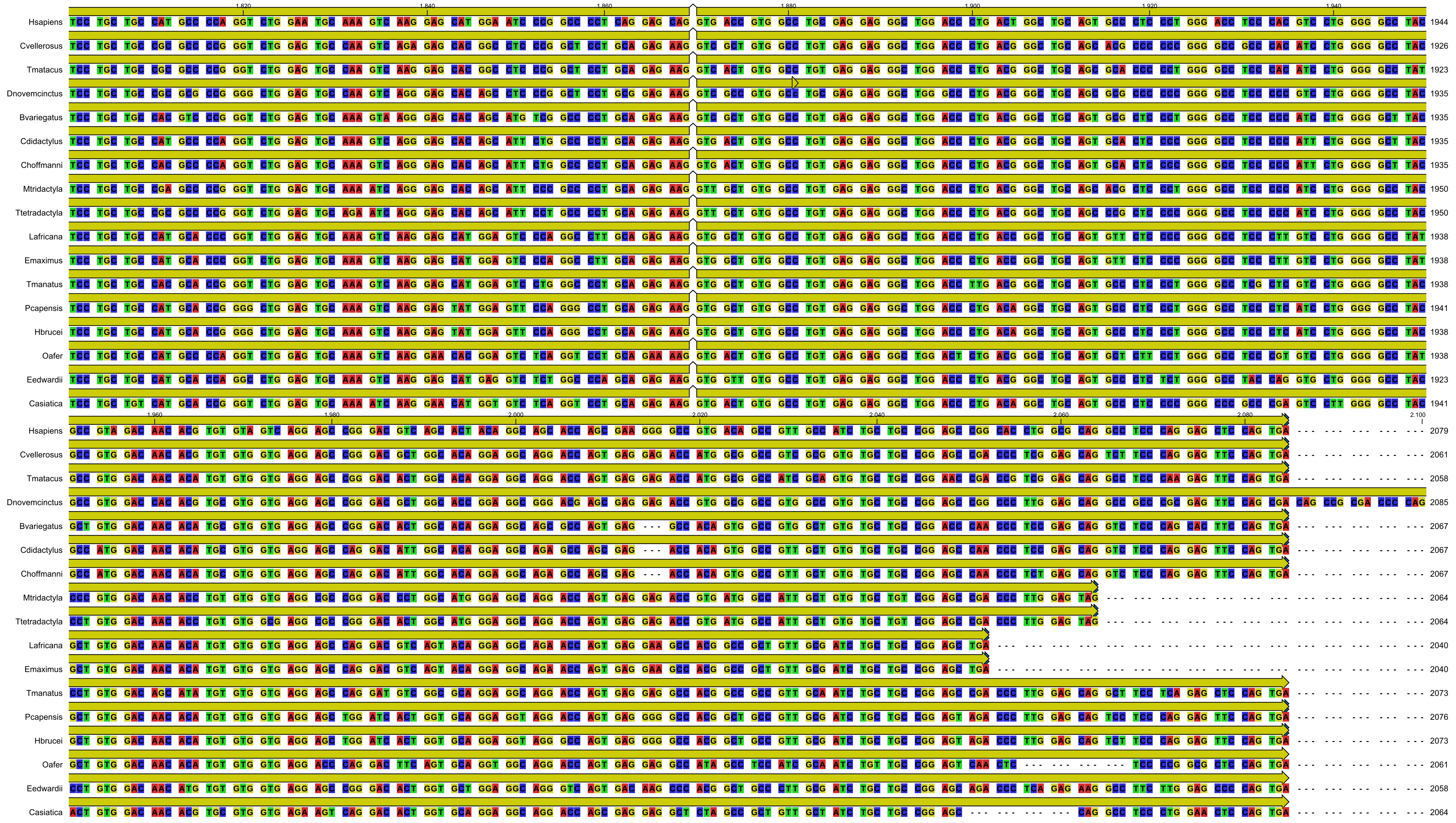
1.820
1.840
1.860
1.880
1.900
1.920
1.940
Hsapiens 1944
Cvellerosus 1926
Tmatacus 1923
Dnovemcinctus 1935
Bvariegatus 1935
Cdidactylus 1935
Choffmanni 1935
Mtridactyla 1950
Ttetradactyla 1950
Lafricana 1938
Emaximus 1938
Tmanatus 1938
Pcapensis 1941
Hbrucei 1938
Oafer 1938
Eedwardii 1923
Casiatica 1941
1.960
1.980
2.000
2.020
2.040
2.060
2.080
2.100
Hsapiens 2079
Cvellerosus 2061
Tmatacus 2058
Dnovemcinctus 2085
Bvariegatus 2067
Cdidactylus 2067
Choffmanni 2067
Mtridactyla 2064
Ttetradactyla 2064
Lafricana 2040
Emaximus 2040
Tmanatus 2073
Pcapensis 2076
Hbrucei 2073
Oafer 2061
Eedwardii 2058
Casiatica 2064

2.120 2.140 2.160 2.180

```
Hsapiens       --- --- --- --- --- --- --- --- --- --- --- --- --- --- --- --- --- --- --- --- --- --- --- --- --- --- --- --- --- --- --- --- --- 2079
Cvellerosus    --- --- --- --- --- --- --- --- --- --- --- --- --- --- --- --- --- --- --- --- --- --- --- --- --- --- --- --- --- --- --- --- --- 2061
Tmatacus       --- --- --- --- --- --- --- --- --- --- --- --- --- --- --- --- --- --- --- --- --- --- --- --- --- --- --- --- --- --- --- --- --- 2058
Dnovemcinctus  GGA CCG AGG CCG GGG TGC GGG CCG GGG CTG GGT GCC GTG GTC CCG TCG GGG GAG CCC TGC AAC GGT TCC GAT TTC CCC TCC TTC AGG TCC CCT CCC TGA 2184
Bvariegatus    --- --- --- --- --- --- --- --- --- --- --- --- --- --- --- --- --- --- --- --- --- --- --- --- --- --- --- --- --- --- --- --- --- 2067
Cdidactylus    --- --- --- --- --- --- --- --- --- --- --- --- --- --- --- --- --- --- --- --- --- --- --- --- --- --- --- --- --- --- --- --- --- 2067
Choffmanni     --- --- --- --- --- --- --- --- --- --- --- --- --- --- --- --- --- --- --- --- --- --- --- --- --- --- --- --- --- --- --- --- --- 2067
Mtridactyla    --- --- --- --- --- --- --- --- --- --- --- --- --- --- --- --- --- --- --- --- --- --- --- --- --- --- --- --- --- --- --- --- --- 2064
Ttetradactyla  --- --- --- --- --- --- --- --- --- --- --- --- --- --- --- --- --- --- --- --- --- --- --- --- --- --- --- --- --- --- --- --- --- 2064
Lafricana      --- --- --- --- --- --- --- --- --- --- --- --- --- --- --- --- --- --- --- --- --- --- --- --- --- --- --- --- --- --- --- --- --- 2040
Emaximus       --- --- --- --- --- --- --- --- --- --- --- --- --- --- --- --- --- --- --- --- --- --- --- --- --- --- --- --- --- --- --- --- --- 2040
Tmanatus       --- --- --- --- --- --- --- --- --- --- --- --- --- --- --- --- --- --- --- --- --- --- --- --- --- --- --- --- --- --- --- --- --- 2073
Pcapensis      --- --- --- --- --- --- --- --- --- --- --- --- --- --- --- --- --- --- --- --- --- --- --- --- --- --- --- --- --- --- --- --- --- 2076
Hbrucei        --- --- --- --- --- --- --- --- --- --- --- --- --- --- --- --- --- --- --- --- --- --- --- --- --- --- --- --- --- --- --- --- --- 2073
Oafer          --- --- --- --- --- --- --- --- --- --- --- --- --- --- --- --- --- --- --- --- --- --- --- --- --- --- --- --- --- --- --- --- --- 2061
Eedwardii      --- --- --- --- --- --- --- --- --- --- --- --- --- --- --- --- --- --- --- --- --- --- --- --- --- --- --- --- --- --- --- --- --- 2058
Casiatica      --- --- --- --- --- --- --- --- --- --- --- --- --- --- --- --- --- --- --- --- --- --- --- --- --- --- --- --- --- --- --- --- --- 2064
```

B

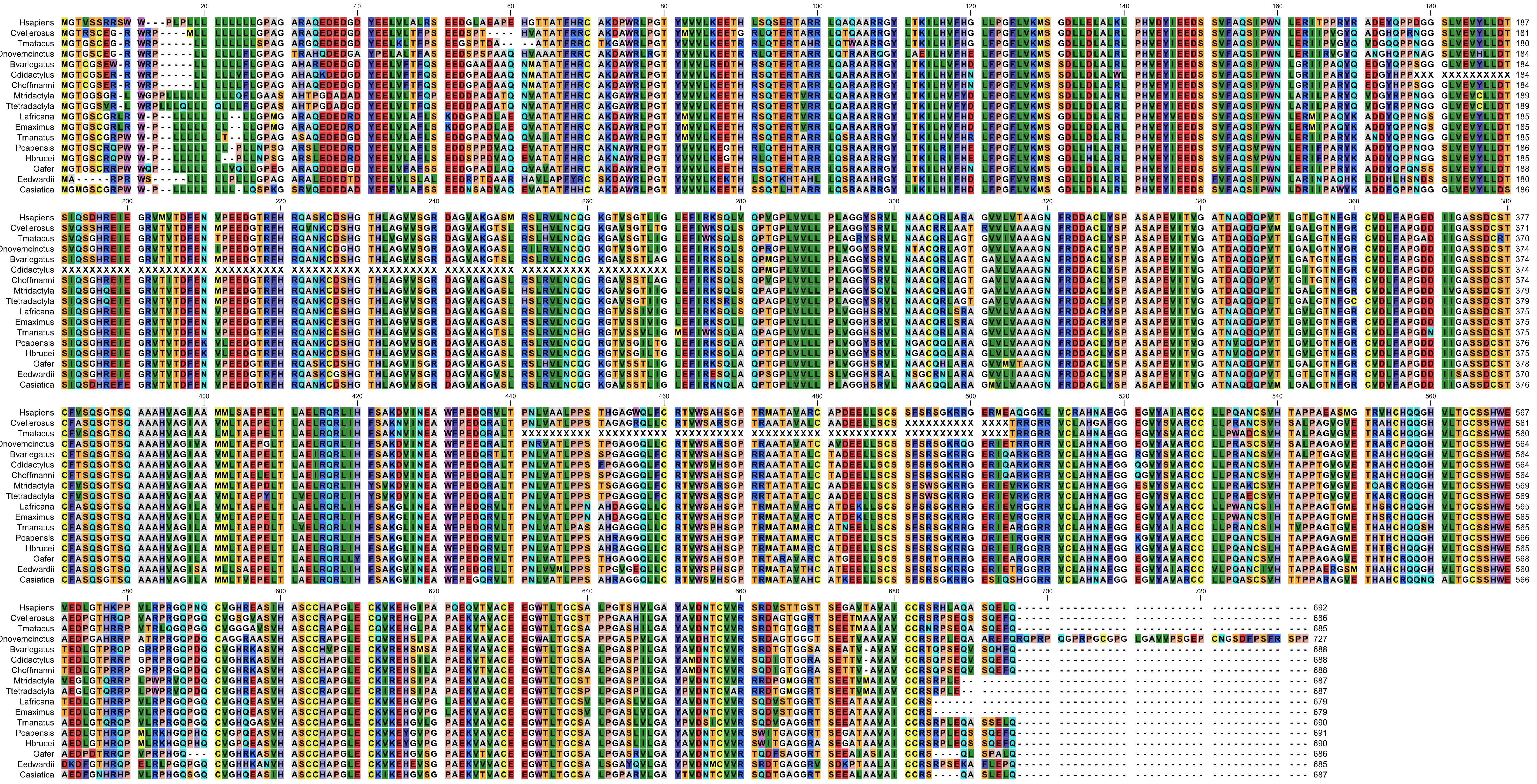
Hsapiens
Cvellerosus
Tmatacus
Dnovemcinctus
Bvariegatus
Cdidactylus
Choffmanni
Mtridactyla
Ttetradactyla
Lafricana
Emaximus
Tmanatus
Pcapensis
Hbrucei
Oafer
Eedwardii
Casiatica
