# Supplementary material for: Patterns and tempo of PCSK9 pseudogenizations suggest an ancient divergence in mammalian cholesterol homeostasis mechanisms
Source: Genetica. 2021 Jan 30;149(1):1–19. doi: 10.1007/s10709-021-00113-x (PMC7929951; doi:10.1007/s10709-021-00113-x)

Supplemental Figure 10.

Comparisons of exon 4 in Leporidae and other Euarchontoglires species. A - cDNA sequences of exons 3 and 4. B - exon 4 and adjacent genomic sequences (the Leporidae -specific mutation which created the new splice acceptor site is boxed in green)

A

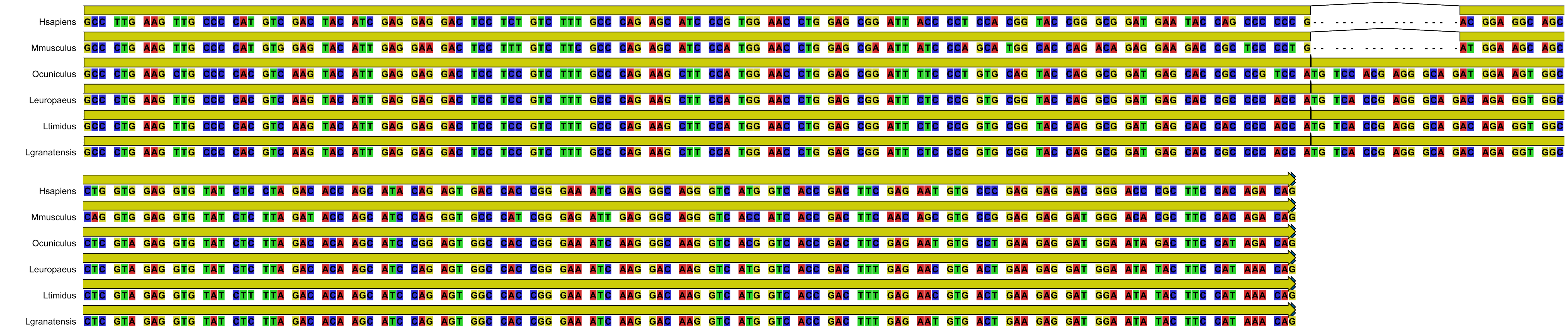

B

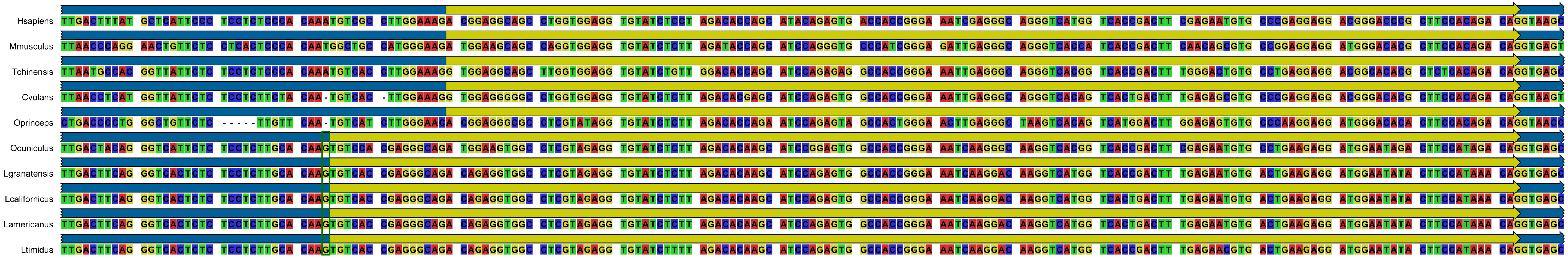

Supplement: Supplementary file 2 — Electronic supplementary material 2 (PDF 840 kb) [file 10709_2021_113_MOESM10_ESM.pdf]
