# Supplementary material for: Patterns and tempo of PCSK9 pseudogenizations suggest an ancient divergence in mammalian cholesterol homeostasis mechanisms
Source: Genetica. 2021 Jan 30;149(1):1–19. doi: 10.1007/s10709-021-00113-x (PMC7929951; doi:10.1007/s10709-021-00113-x)

Supplemental Figure 2.

DNA sequence alignments of the exon11-intron11 and intron11-exon 12 border regions in Xenarthra *vs. H. sapiens*. The 1 bp deletion in exon12 and the 10 bp extension of exon11 present in the reference sequence of *D. novemcinctus* are indicated with red arrowheads

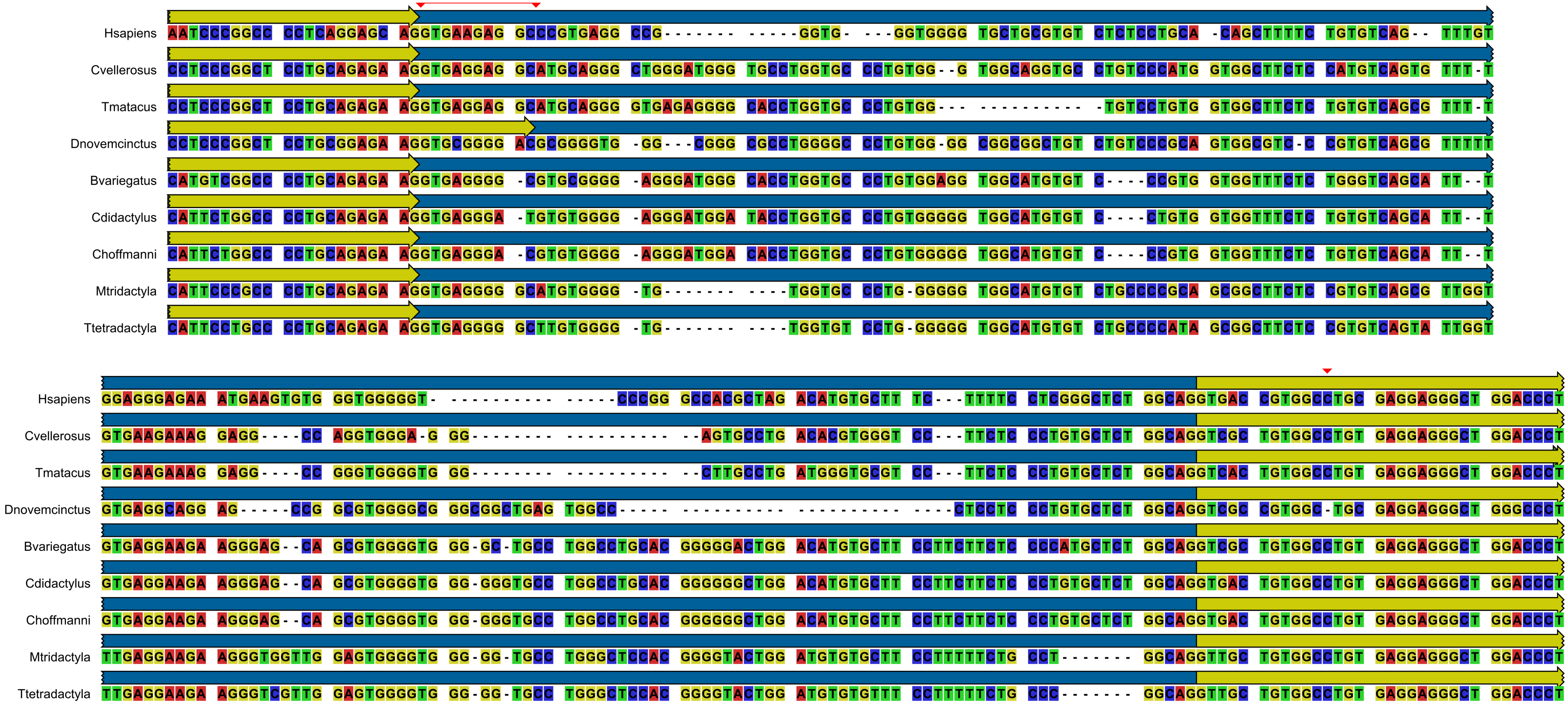

Supplement: Supplementary file 7 — Electronic supplementary material 7 (PDF 596 kb) [file 10709_2021_113_MOESM2_ESM.pdf]
