# Supplementary material for: Patterns and tempo of PCSK9 pseudogenizations suggest an ancient divergence in mammalian cholesterol homeostasis mechanisms
Source: Genetica. 2021 Jan 30;149(1):1–19. doi: 10.1007/s10709-021-00113-x (PMC7929951; doi:10.1007/s10709-021-00113-x)

Supplemental Figure 3.

Sequence alignment of the regions surrounding the intron3-exon4 border in Suidae, other Laurasiatheria and *H. sapiens*

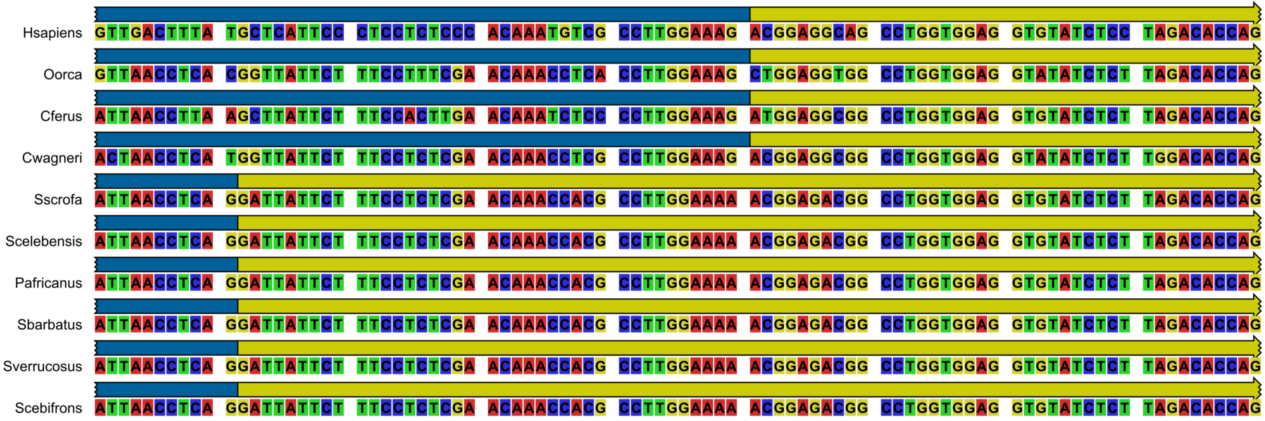

Supplement: Supplementary file 8 — Electronic supplementary material 8 (PDF 957 kb) [file 10709_2021_113_MOESM3_ESM.pdf]
