# Supplementary material for: Patterns and tempo of PCSK9 pseudogenizations suggest an ancient divergence in mammalian cholesterol homeostasis mechanisms
Source: Genetica. 2021 Jan 30;149(1):1–19. doi: 10.1007/s10709-021-00113-x (PMC7929951; doi:10.1007/s10709-021-00113-x)

Supplemental Figure 4.

Alignment of the PCSK9 protein sequences from 35 of the 36 species of Laurasiatheria found to have a functional gene (the S. barbatus was rather incomplete). Residues not determined due to incomplete DNA sequence are indicated as X; arrowheads indicate residues crucial for catalysis (red) and the 25 conserved cysteines (black). Protein domains are shaded with different colors.

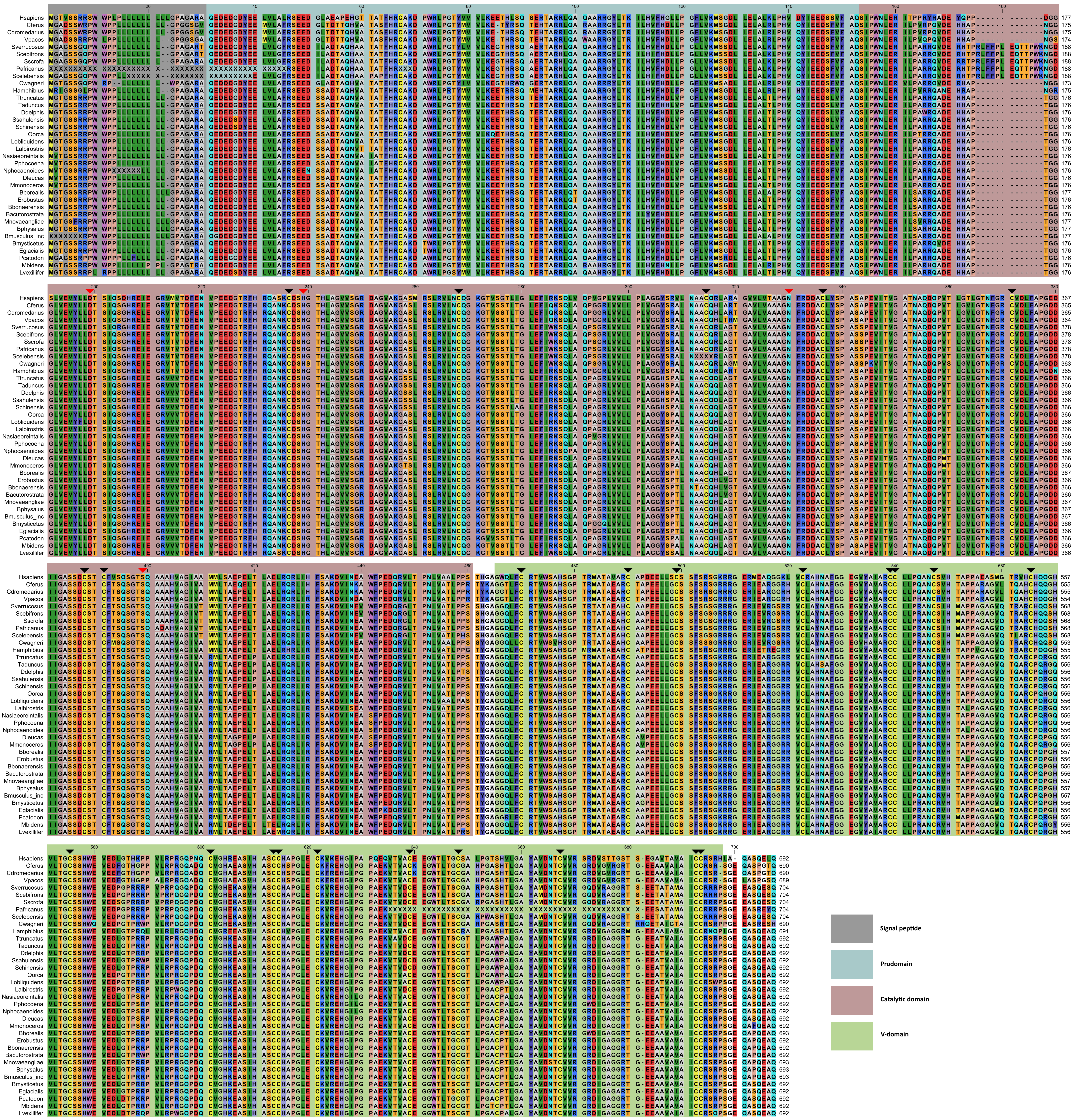

Supplement: Supplementary file 9 — Electronic supplementary material 9 (PDF 7164 kb) [file 10709_2021_113_MOESM4_ESM.pdf]
