# Supplementary material for: Patterns and tempo of PCSK9 pseudogenizations suggest an ancient divergence in mammalian cholesterol homeostasis mechanisms
Source: Genetica. 2021 Jan 30;149(1):1–19. doi: 10.1007/s10709-021-00113-x (PMC7929951; doi:10.1007/s10709-021-00113-x)
Supplement: Supplementary file 10 — Electronic supplementary material 10 (PDF 6507 kb) [file 10709_2021_113_MOESM5_ESM.pdf]

Supplemental Figure 5.

Sequence alignments of the breakpoint regions in Laurasiatheria species with a *PCSK9* pseudogene vs O. Orca sequences. A - 15.4 kb deletion; B - 1.4 kb deletion

A

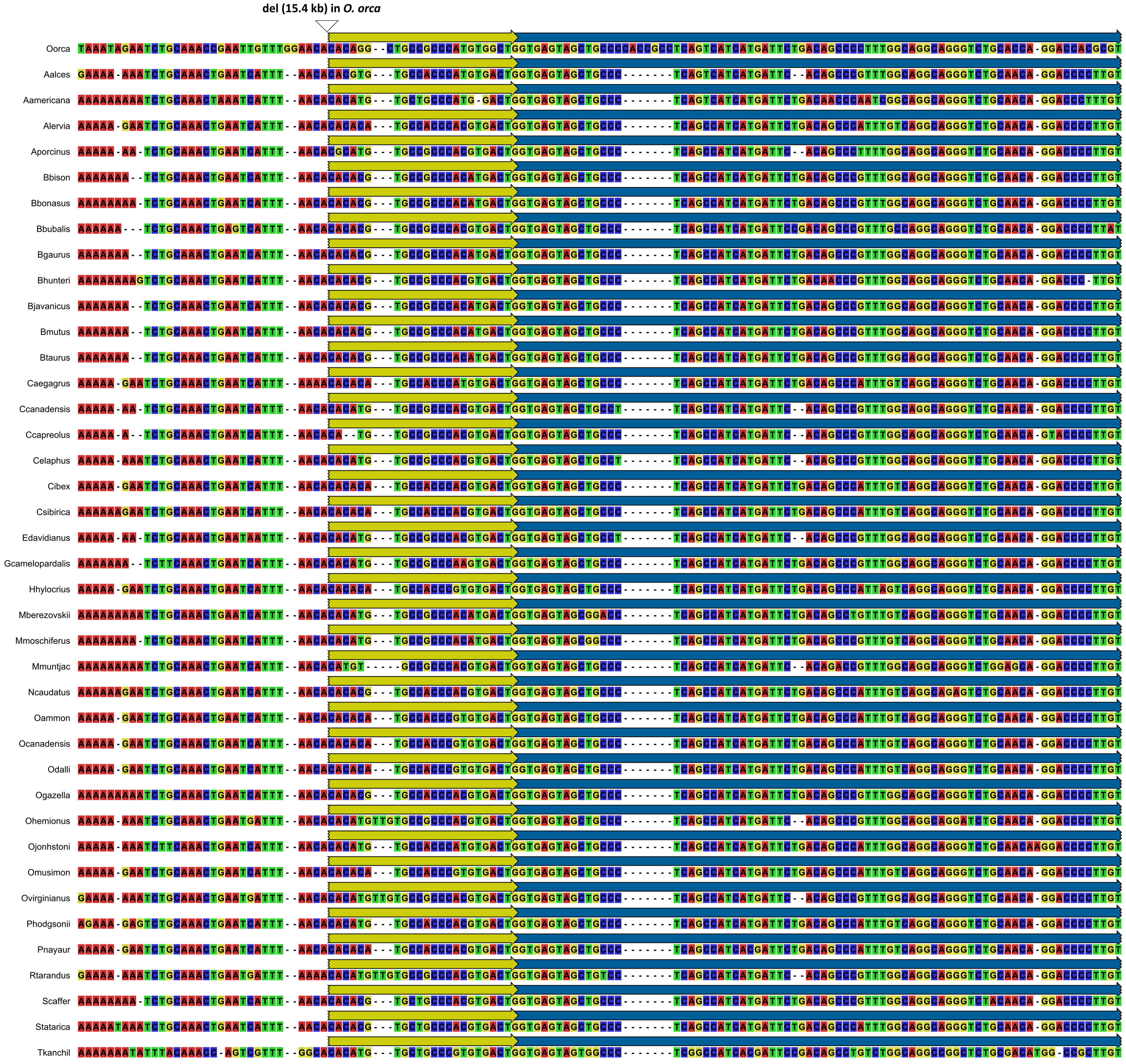

B

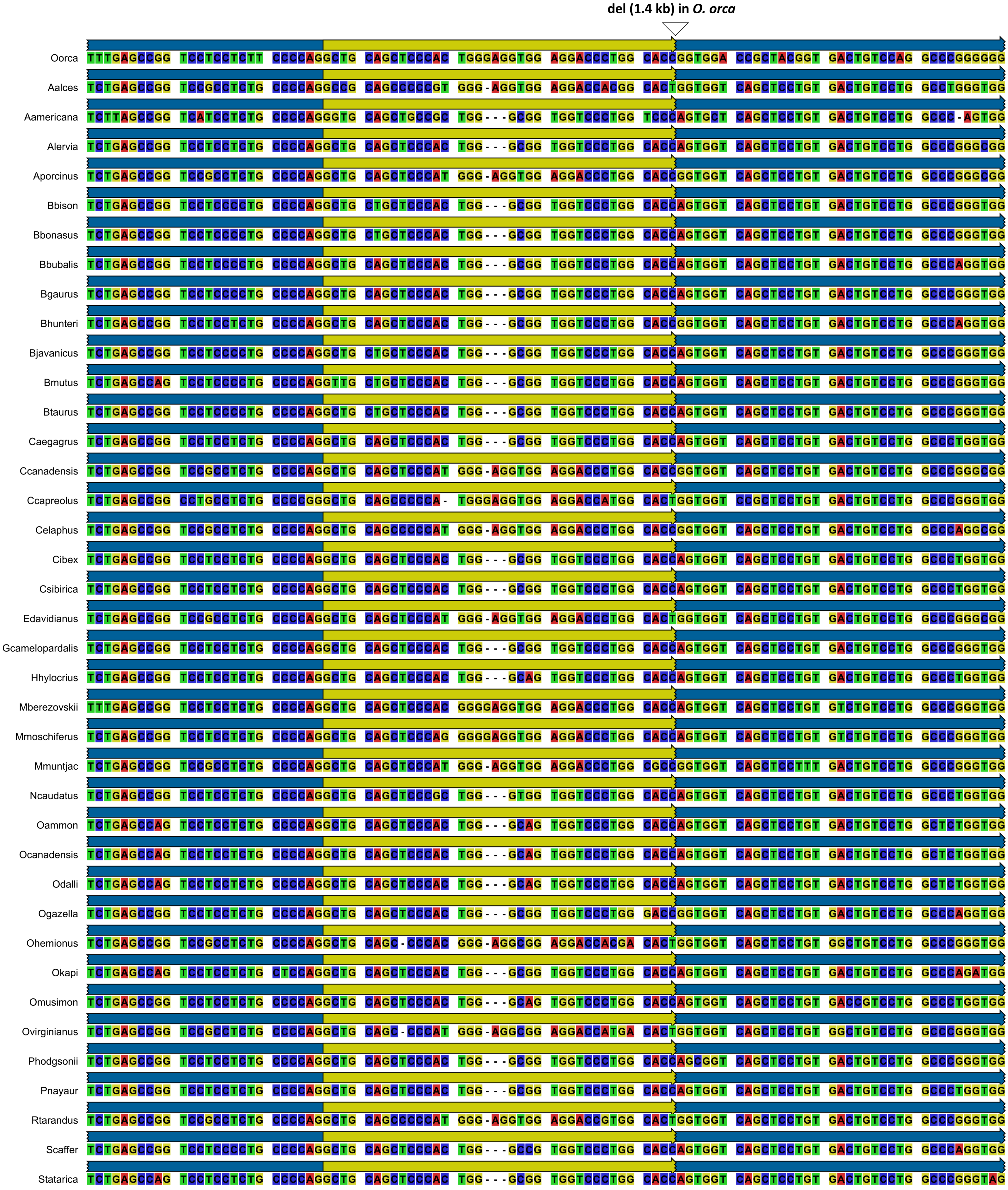
del (1.4 kb) in O. orca
Oorca
Aalces
Aamericana
Alervia
Aporcinus
Bbison
Bbonasus
Bbubalis
Bgaurus
Bhunteri
Bjavanicus
Bmutus
Btaurus
Caegagrus
Ccanadensis
Ccapreolus
Celaphus
Cibex
Csibirica
Edavidianus
Gcamelopardalis
Hhylocrius
Mberezovskii
Mmoschiferus
Mmuntjac
Ncaudatus
Oammon
Ocanadensis
Odalli
Ogazella
Ohemionus
Okapi
Omusimon
Ovirginianus
Phodgsonii
Pnayaur
Rtarandus
Scaffer
Statarica
