# Supplementary material for: Patterns and tempo of PCSK9 pseudogenizations suggest an ancient divergence in mammalian cholesterol homeostasis mechanisms
Source: Genetica. 2021 Jan 30;149(1):1–19. doi: 10.1007/s10709-021-00113-x (PMC7929951; doi:10.1007/s10709-021-00113-x)

Supplemental Figure 6.

Alignment of the Talpidae protein sequences vs. O. Orca and H. sapiens. Arrowheads indicate residues crucial for catalysis (red) and the conserved cysteines (black). Protein domains are shaded with different colors.

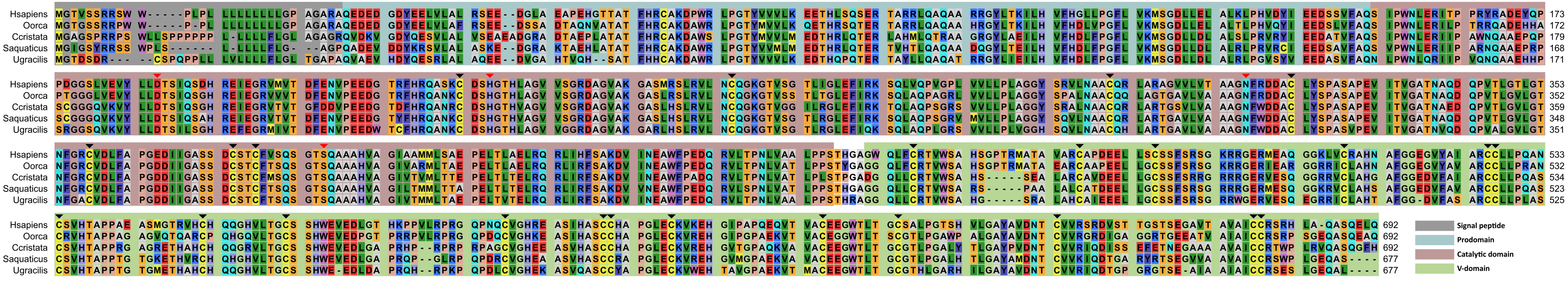

Supplement: Supplementary file 11 — Electronic supplementary material 11 (PDF 3684 kb) [file 10709_2021_113_MOESM6_ESM.pdf]
