# Supplementary material for: Patterns and tempo of PCSK9 pseudogenizations suggest an ancient divergence in mammalian cholesterol homeostasis mechanisms
Source: Genetica. 2021 Jan 30;149(1):1–19. doi: 10.1007/s10709-021-00113-x (PMC7929951; doi:10.1007/s10709-021-00113-x)

Supplemental Figure 7.

Alignments of recognizable exonic sequences of *S. paradoxus*. A - Exon 2 vs *O. orca* and Talpidae; exon 12 (non-coding sequences) vs *O. orca* (no homology against Talpidae 3'-UTR was found)

A

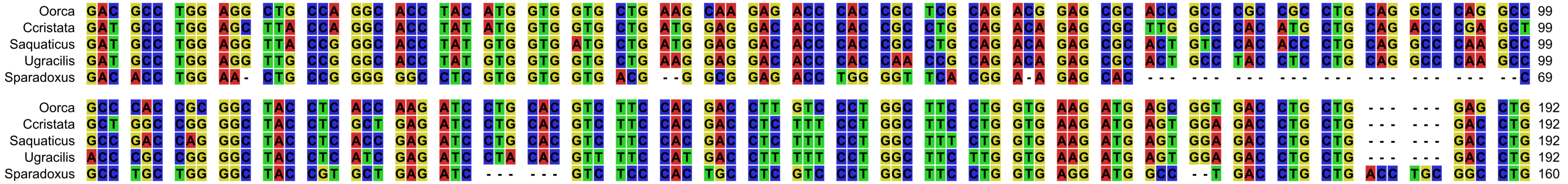

B

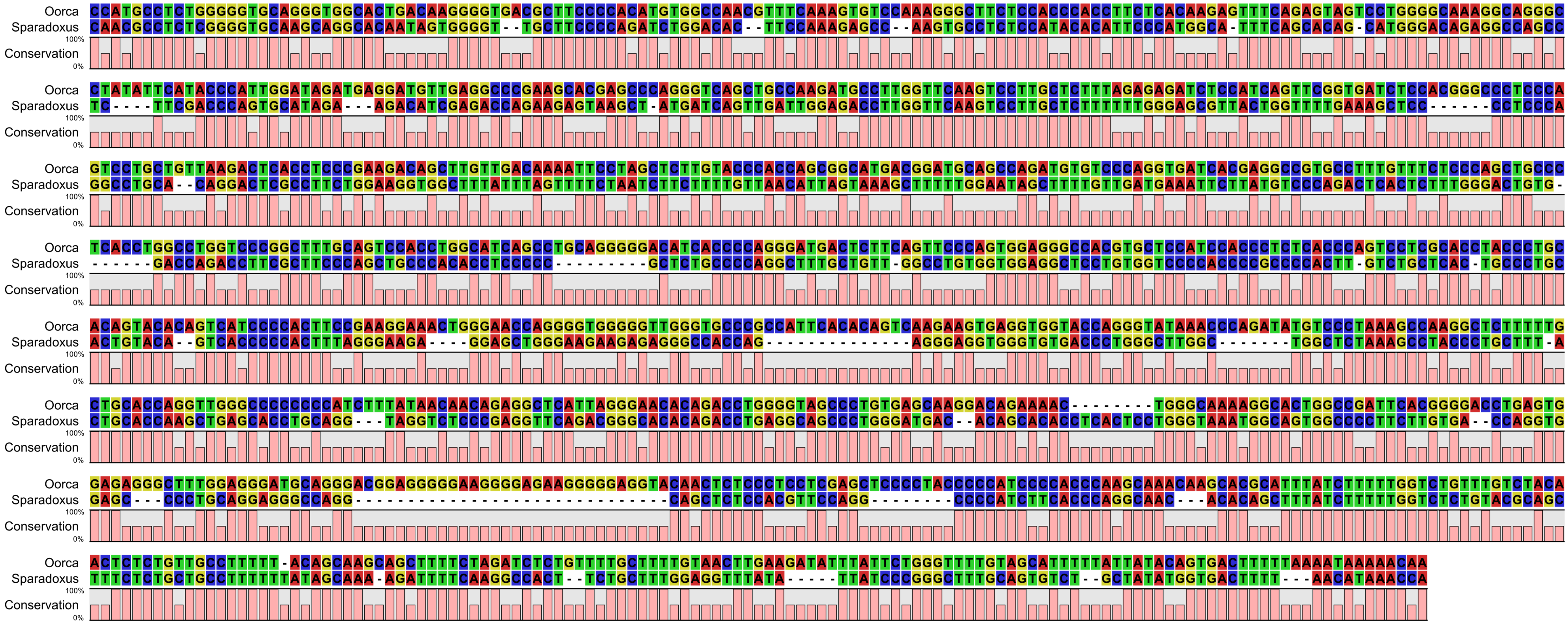

Supplement: Supplementary file 12 — Electronic supplementary material 12 (PDF 3278 kb) [file 10709_2021_113_MOESM7_ESM.pdf]
