# Supplementary material for: Patterns and tempo of PCSK9 pseudogenizations suggest an ancient divergence in mammalian cholesterol homeostasis mechanisms
Source: Genetica. 2021 Jan 30;149(1):1–19. doi: 10.1007/s10709-021-00113-x (PMC7929951; doi:10.1007/s10709-021-00113-x)

Supplemental Figure 8.

Sequence alignments of Dipodidae *vs* Pedetidae, Spalacidae and Rhizomyidae. A - DNA sequences of exons 6 to 12 (blue vertical bars mark exon limits; red horizontal bars indicate frameshifting indels). B - Protein sequences (red arrowheads indicate actual or potencial protein inactivating alterations; X indicates residues not determined due to incomplete DNA sequence)

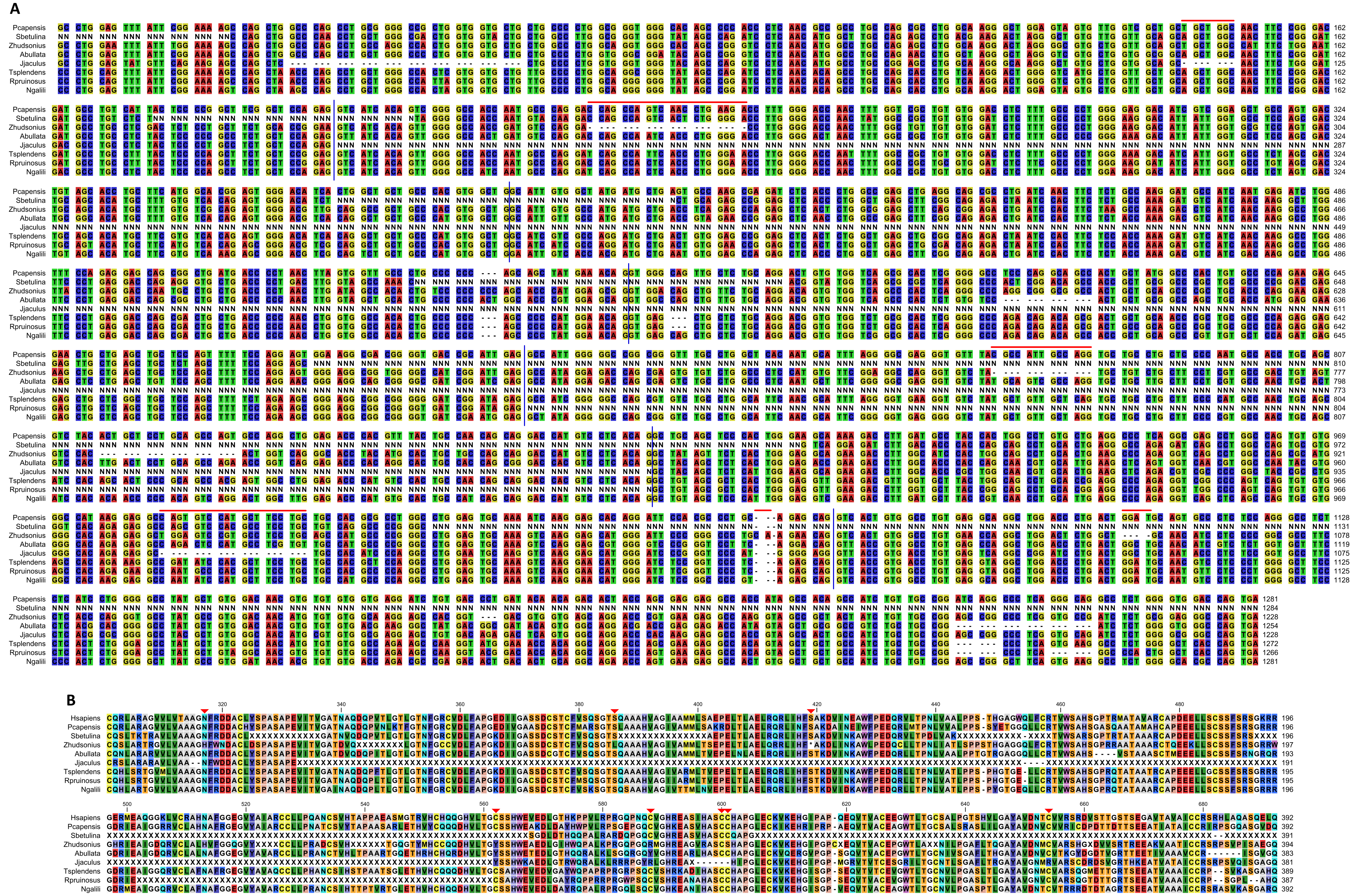

Supplement: Supplementary file 13 — Electronic supplementary material 13 (PDF 3053 kb) [file 10709_2021_113_MOESM8_ESM.pdf]
