# Supplementary material for: Patterns and tempo of PCSK9 pseudogenizations suggest an ancient divergence in mammalian cholesterol homeostasis mechanisms
Source: Genetica. 2021 Jan 30;149(1):1–19. doi: 10.1007/s10709-021-00113-x (PMC7929951; doi:10.1007/s10709-021-00113-x)

Supplemental Figure 9.

Alignment of sequences surrounding the exon3-intron3 border in Cricetidae, *M. musculus* (Muridae) and *C. gambianus* (Nesomyidae). Exons in yellow; introns in blue. Mutations affecting one of the two possible splice donor sites are boxed in red

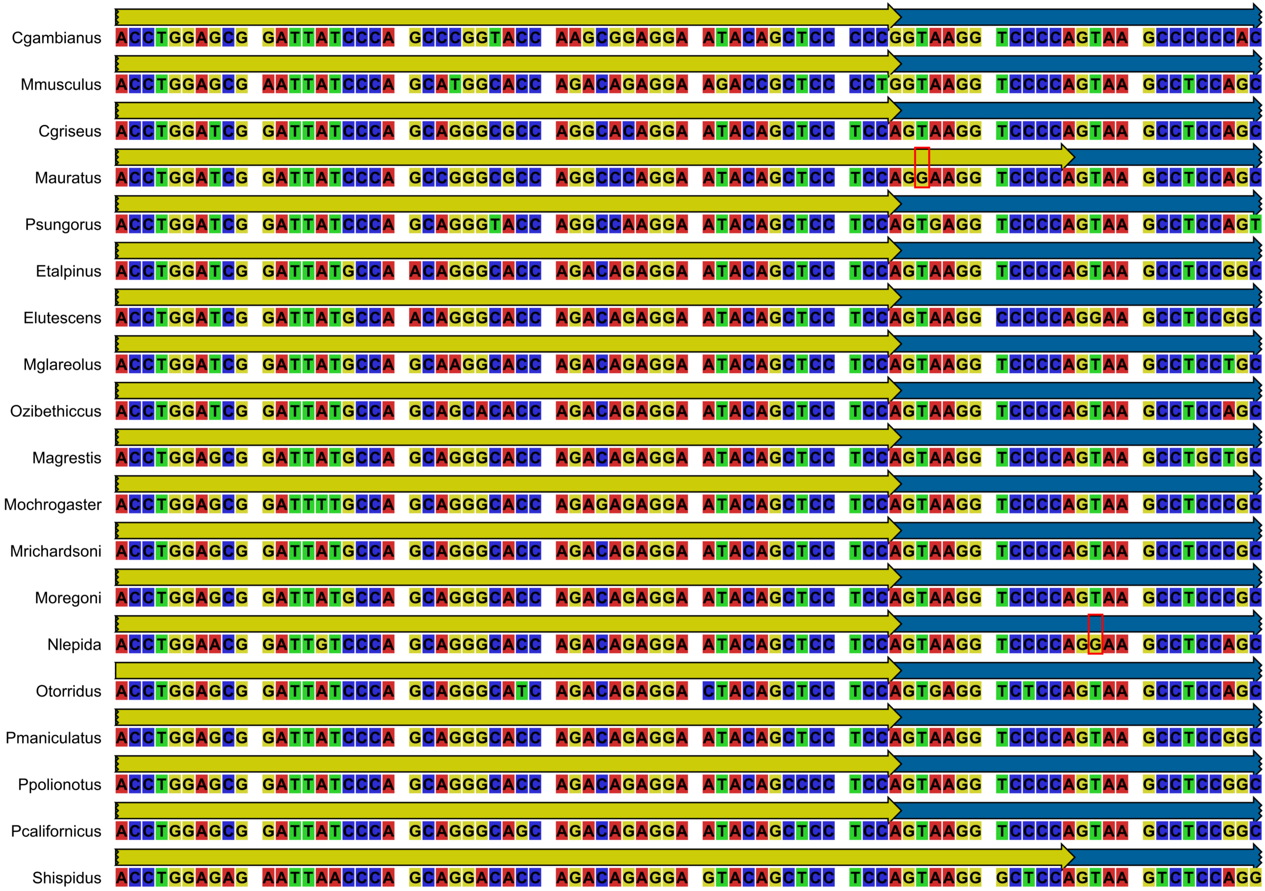

Supplement: Supplementary file 14 — Electronic supplementary material 14 (PDF 1901 kb) [file 10709_2021_113_MOESM9_ESM.pdf]
